# Design, Synthesis, and Study of Protective Activity Against Stroke for Novel Water-Soluble Aldehyde Dehydrogenase 2 Activators

**DOI:** 10.3390/molecules30142924

**Published:** 2025-07-10

**Authors:** Fengping Zhao, Zhenming Yu, Wei Tian, Xinhui Huang, Qingsen Zhang, Ruolan Zhou, Jian Hu, Shichong Yu, Xin Chen, Canhui Zheng

**Affiliations:** 1 School of Life Science and Technology, Wuhan Polytechnic University, Wuhan 430023, China; 2 The Center for Basic Research and Innovation of Medicine and Pharmacy (MOE), School of Pharmacy, Naval Medical University (Second Military Medical University), Shanghai 200433, China; 3 General Hospital of Central Theater Commond, Wuhan 430070, China

**Keywords:** stroke, cerebral infarction, aldehyde dehydrogenase 2, activators, water-soluble

## Abstract

Stroke poses a serious threat to human health, while there are very few drugs that can directly alleviate ischemia/reperfusion injury and improve the prognosis. Studies have shown that small-molecule activators of aldehyde dehydrogenase 2 (ALDH2) have the potential to become novel therapeutic drugs for ischemic stroke. In this study, through the systematic structural optimization of novel *N*-benzylaniline-based ALDH2 activators obtained from our previous virtual screening, ALDH2 activators with improved water solubility and activity were obtained. Among them, compound **D10** exhibits the best activity, with a maximum activation fold reaching 114% relative to Alda-1. And the water solubility of its hydrochloride salt **D27** was increased by more than 200-fold. The intravenous injection of this compound can significantly reduce the infarct area in the rat model of cerebral infarction compared with the model group. This study lays a good foundation for the future research on ALDH2 activators used in the treatment of stroke.

## 1. Introduction

Currently, the number of prevalent stroke cases worldwide exceeds 100 million, among which ischemic stroke (IS) accounts for approximately 80%. IS is characterized by high mortality and disability rates [1,2,3,4,5,6]. At present, reperfusion therapy is the main treatment for IS. During the treatment, cerebral ischemia/reperfusion (I/R) is unavoidable. It ultimately exacerbates injury by inducing the opening of mitochondrial permeability transition pores (MPTPs) to cause cell lysis and death, which is an important cause of poor prognosis. However, there are currently few drugs that can directly alleviate I/R injury and improve the prognosis [7,8]. Therefore, there is an urgent need for research on novel drugs that can reduce cerebral I/R injury and, thus, improve the prognosis of IS. In recent years, increasing studies have found that aldehyde dehydrogenase 2 (ALDH2) is closely associated with the risk and prognosis of IS, making ALDH2 a promising target [9,10,11,12,13,14,15].

Studies in recent years have shown that ALDH2 is responsible for the metabolism of toxic aldehydes such as 4-hydroxy-2-nonenal (4-HNE) generated under endogenous oxidative stress. Therefore, ALDH2 is an important part of the body’s defense system against oxidative stress damage [10,16]. It is worth noting that a high proportion of the East Asian population carries a unique inactivated variant, ALDH2*2, with a prevalence rate of 35–45% in the East Asian population [17,18]. The enzymatic activity of individuals homozygous for ALDH2*2 is only 1–4% of that of individuals without the mutation, and the enzymatic activity of heterozygous individuals is only 20–40% of that [19,20]. During IS, toxic aldehydes such as 4-HNE and malondialdehyde produced by oxidative stress can cause cell damage through multiple pathways, impairing various important functions of the brain tissue and exacerbating cerebral I/R injury [10,21,22]. Conversely, the overexpression of ALDH2 or the administration of ALDH2 activators can alleviate I/R injury and, thus, improve the prognosis [21,22,23,24], including improving neurological function or reducing cerebral edema after cerebral I/R injury [25]. Therefore, small-molecule ALDH2 activators have the potential to become novel therapeutic drugs for IS.

**Figure 1:**
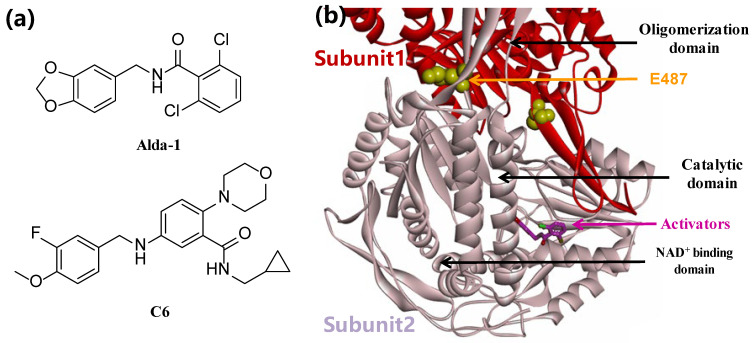
(**a**) Structures of representative activators; and (**b**) topological structure of ALDH2. The protein structure in the figure is shown using a ribbon model, with the two subunits represented in pink and red, respectively. Three regions of each subunit are labeled. The mutated amino acids in the ALDH2 variant are shown using a yellow CPK model, and the small-molecule activators are demonstrated using a purple stick model.

The research on small-molecule ALDH2 activators is still in its infancy. Previously, Alda-1, a small-molecule ALDH2 activator tool drug discovered through high-throughput experimental screening, was reported, as well as its derivatives (Figure 1a) [23,26,27,28]. Studies have found that Alda-1 can not only significantly increase the activity of the inactivated variant ALDH2*2 but also enhance the activity of the wild-type ALDH2. Different levels of research conducted by researchers using this tool drug suggest that using small-molecule ALDH2 activators as novel therapeutic drugs for IS is feasible [16,23]. Our research group previously obtained a novel class of *N*-benzylaniline-based ALDH2 activators through virtual screening and preliminary structural optimization (Figure 1a), which have a protective effect on animal models of cerebral infarction [29]. However, due to the unsatisfactory physicochemical properties of these two reported classes of activators, the protective effect can only be observed by intracerebroventricular injection in a rat model of cerebral I/R injury, and cannot be achieved by conventional intravenous injection.

Aiming at these existing problems, this study systematically optimized *N*-benzylaniline-based ALDH2 activators based on their binding mode with ALDH2, obtained water-soluble derivatives, and evaluated their ALDH2 activation activity, as well as their protective effect on animal models of cerebral infarction under conventional injection methods.

## 2. Results and Discussion

### 2.1. Obtaining of Novel N-Benzylaniline-Based ALDH2 Activators

**Figure 2:**
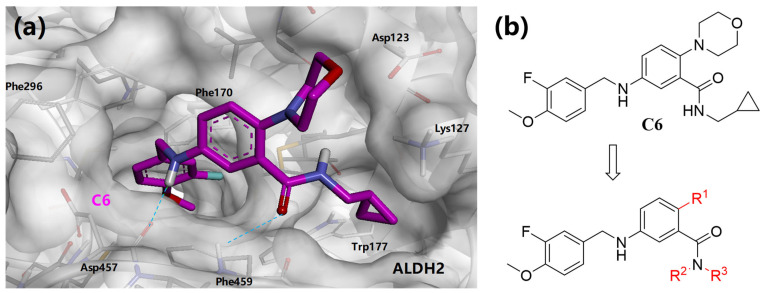
(**a**) Binding mode of *N*-benzylaniline-based activator **C6** with ALDH2. The ALDH2 protein structure template is derived from the crystal structure (PDB ID: 3INJ). The protein is shown as a gray ribbon and a solvent-accessible surface, and the residues important for binding are shown as gray stick models. The activator is shown as a purple stick model, and the hydrogen bonds are shown as blue dashed lines. (**b**) The design of novel *N*-benzylaniline-based ALDH2 activators.

As shown in Figure 1b, ALDH2 exists as two dimers. Each subunit contains a catalytic domain, a coenzyme NAD^+^ binding domain, and an oligomerization domain. The ALDH2*2 variant arises from an amino acid mutation (E487K), which affects the conformations of the catalytic domain and the coenzyme binding domain of the enzyme through an allosteric effect, ultimately causing a significant reduction in enzymatic activity [20,30]. The activator binds to the channel from the catalytic site to the protein exterior and enhances enzymatic activity by stabilizing the protein conformation through an allosteric effect [31]. Docking studies revealed the binding mode of the lead compound **C6** to ALDH2, as shown in Figure 2a and Table S1. This binding mode rationalizes the preliminary structure–activity relationships derived from prior structural optimization, and provides a basis for the further optimization and design. The benzylamino moiety of the lead compound inserts into the enzyme interior: its benzene ring forms *π–π* interactions with the planar side chains of Phe296, Phe170, and Phe459, while the amino group forms a hydrogen bond with the backbone of Asp457. This moiety is critical for binding and was retained in the subsequent structural modification. The alkyl-substituted amide group part of the compound binds to a hydrophobic pocket on the protein surface, and its carbonyl group forms a hydrogen bond with the backbone of Phe459. The morpholine ring binds to another polar pocket on the protein surface, composed of residues such as Asp123. These two regions serve as key sites for further structural modification. The general structure of target compounds, designed by introducing various basic and polar groups at these two positions, is shown in Figure 2b and Table S1.

First, we introduced the amino group at different positions of the alkyl-substituted amide moiety of the lead compound, obtaining compounds **D1**–**D4**. We used the previously established method to evaluate the in vitro ALDH2 activation activity of these compounds (Table 1). The maximum activation fold was used as the primary indicator, with activity values calibrated against Alda-1. The maximum activation folds of Alda-1 ranged from 1.98 to 2.44 (designated as 100%). The test results showed that compounds **D1** and **D2** exhibited activity comparable to that of the lead compound, likely due to their amino groups facing the solvent. However, compound **D4** showed a significant activity decrease, possibly owing to the excessively small volume of its alkyl group, consistent with the previous structure–activity relationship studies indicating that three to four carbon atoms at this position are the optimal volume [27].

Given that the morpholine ring of the lead compound binds to a polar pocket on the protein surface comprising residues such as Asp123, we focused on modifying this region, introducing different types and improving the activity. We replaced morpholine with nitrogen-substituted piperazine, aminopiperidines, open-chain amino aliphatic chains, and piperidine rings, yielding compounds **D5**–**D9**. ALDH2 activation assays showed that compounds **D5** (morpholine replaced with unsubstituted piperazine) and **D9** (replaced with *N*-aminoethyl-*N*-methyl-substituted amino groups) had activities comparable to the lead compound. We further extended the basic group in this region by inserting methylene or carbonyl groups, generating **D10**–**D17**. Compound **D14** (morpholine replaced with a *N*-aminoethyl-*N*-methyl-substituted aminomethyl group) showed a significant activity decrease, whereas compound **D10** (replaced with a piperazine methylene group) increased activity.

Finally, we replaced the morpholine with piperidine rings and differently substituted aromatic pyridine/pyrazole rings, obtaining compounds **D18**–**D22**. Additionally, using **D5** as a template, we modified the cyclopropyl methylene group to generate compounds **D23**–**D26**. Compound **D18** (morpholine replaced with a piperidine group having a distal nitrogen atom) showed significantly reduced activity, while tests indicated that compound **D25** had activity comparable to the lead compound.

**Figure 3:**
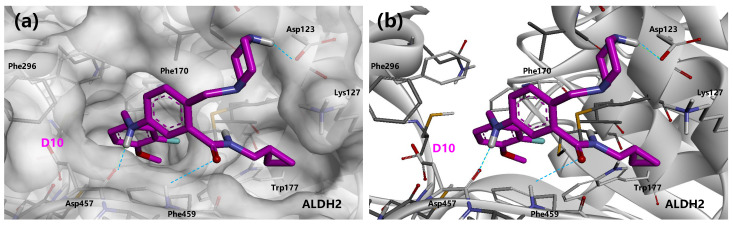
Binding mode of *N*-benzylaniline-based activator **D10** with ALDH2 shown as solvent-accessible surface models (**a**) or gray ribbon models (**b**). The ALDH2 protein structure template is derived from the crystal structure (PDB ID: 3INJ). The residues important for binding are shown as gray stick models. The activators are shown as purple stick models, and the hydrogen bonds are shown as blue dashed lines.

Based on these results, introducing amino or other basic groups into either the alkyl-substituted amide group moiety or morpholine ring of the lead compound impacts activity sensitively. Activity is maintained or improved only when introduced at specific positions. Among these, compound **D10** showed the highest activity, with a maximum activation fold of 118% relative to Alda-1. Its EC_50_ value was evaluated further as 16.8 µM. Docking-derived binding modes (Figure 3 and Table S1) revealed that the piperazine group forms a hydrogen bond with Asp123, likely contributing to its enhanced activity.

### 2.2. Synthesis of Novel N-Benzylaniline-Based ALDH2 Activators

**Scheme 1:**
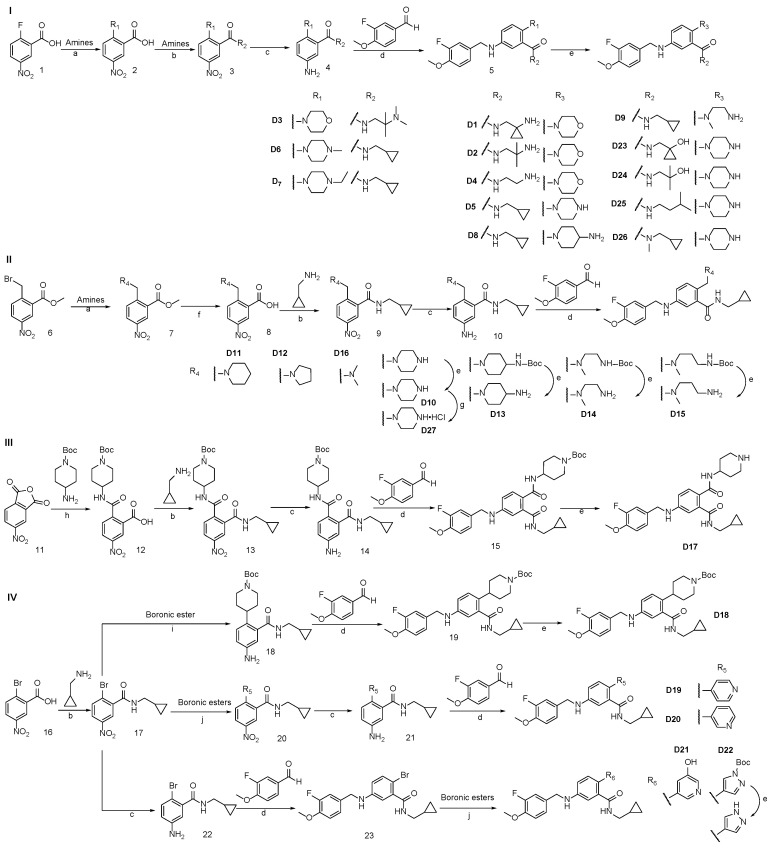
Four synthetic routes I–IV of novel *N*-benzylaniline-based ALDH2 activators. Reagents and conditions: (a) DMSO, K_2_CO_3_, 80 °C, reflux, 1–4.5 h, 79–99%; (b) DMAP, EDCI, DCM, r. t. 1.5–6 h, 67–95%; (c) EtOH, NH_4_Cl, Fe, H_2_O, 80 °C, reflux, 1–3 h, 71–97%; (d) DCE, NaBH(OAc)_3_, 80 °C reflux, r. t., 2–12 h, 59–95%; (e) DCM, TFA, r. t., 0.5–2.5 h, 73–90%; (f) NaOH, MeOH, THF, H_2_O, 1–2 h, 91%; (g) conc-HCl, EA, 1.5 h, r. t., 69%; (h) HATU, DIPEA, DMF, 80 °C, 12 h, 42.8%; (i) PdXPhosG_2_, Pd/C, K_3_PO_4_, Dioxane/H_2_O (4:1), 80 °C, 4 h; then MeOH, NH_4_COOH, r. t. to 60 °C, 16 h, 75%; and (j) Pd(dppf)Cl_2_, K_2_CO_3_, Dioxane/H_2_O (3:1), 100 °C, 12 h, ~70%.

The synthesis of target compounds follows four synthetic routes (Scheme 1), primarily comprising four steps: the nucleophilic substitution or coupling reactions of halogenated compounds; condensation reactions between acids and amines; the iron-mediated reduction of nitro groups; and the reductive amination of amines with aldehydes.

Compounds **D1**–**D9** and **D23**–**D26** were synthesized via the route as follows: Starting with 2-fluoro-5-nitrobenzoic acid, intermediate **2** is formed via nucleophilic substitution with different amines. This is followed by condensation with another amine to generate intermediate **3**. The nitro group on this intermediate is then reduced to the corresponding amine using iron. Finally, the benzylaniline core scaffold is constructed through the reductive amination of the amine with an aldehyde, yielding compounds **D3**, **D6,** and **D7**. Compounds **D1**, **D2**, **D4**, **D5**, **D8**, **D9,** and **D23**–**D26** are obtained by further removing the Boc protecting group after the core scaffold is constructed.

Compounds **D10**–**D16** were synthesized via Route II, similar to Route I. Considering the cost of the starting materials, methyl 2-(bromomethyl)-5-nitrobenzoate was used in this route instead of the corresponding carboxylic acid. Consequently, intermediate **7** obtained after substitution must undergo hydrolysis before the acid–amine condensation step. In addition, compounds **D10** and **D13**–**D15** also require Boc deprotection after constructing the benzylaniline core scaffold. The hydrochloride salt **D27** was synthesized from **D10** by stirring with concentrated hydrochloride. Compound **D17** was synthesized via Route III, also similar to Route I, with 5-nitroisobenzofuran-1,3-dione as the starting material.

Compounds **D18**–**D22** were synthesized via Route IV, in which the R4 moiety was constructed via a coupling reaction. Starting with 2-bromo-5-nitrobenzoic acid, intermediate **17** is formed by condensation with cyclopropanemethylamine. Subsequently, coupling and reduction reactions are sequentially performed in the same reaction vessel, followed by steps identical to Route I to yield **D18**. For **D19** and **D20**, the coupling and reduction steps are separated. Compounds **D12** and **D22** were obtained through different chemical synthesis sequences, due to the commercial availability of intermediates.

### 2.3. Evaluation of the Water Solubility of Novel ALDH2 Activators and Their Protective Effects on Animal Models of Cerebral Infarction

**Figure 4:**
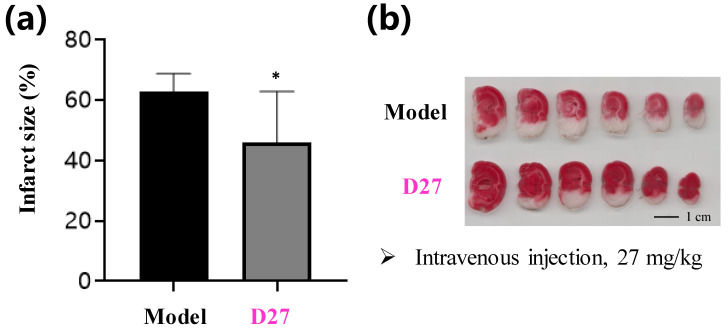
(**a**) Protective effect of **D27** against cerebral infarction in a middle cerebral artery occlusion (MCAO) rat model. The infarct area in MCAO rats is shown as mean ± SD, * *p* < 0.05, n = 6. (**b**) Representative brain tissue sections of rats in the **D27** group and the model group. The ischemic infarct region is white.

Through the above structural optimization, we obtained compound **D10**, whose features improved the activity and the introduction of a basic piperazine moiety. We synthesized its hydrochloride salt **D27** and determined its water solubility using the HPLC method. The solubility is 6.7 mg/mL, which is significantly higher than that of the lead compound **C6**. The solubility of **C6** in water is 0.024 mg/mL, similar to that of Alda-1. Therefore, this compound can be formulated into an aqueous solution, and its protective efficacy on a rat model of cerebral infarction was evaluated via intravenous injection (Figure 4).

We performed the middle cerebral artery occlusion (MCAO) surgery on rats. After 15 min of ischemia, the sample was injected via the tail vein. After 2 h of ischemia, reperfusion was carried out for 24 h. After sacrificing the rats, brain tissues were collected for 2,3,5-triphenyltetrazolium chloride (TTC) staining. The results showed that the average infarct area of the model group was 63%, while that in the sample-treated group dropped significantly to 46%. Although the effect of compound **D27** is moderate, it can be administered via peripheral intravenous injection and exhibits protective effects against cerebral infarction. It represents a class of ALDH2 activators worthy of further investigation.

## 3. Materials and Methods

### 3.1. Molecular Docking

The crystal structure of the ALDH2-activator complex (PDB ID: 3INJ) served as the template for molecular docking. Molecular docking was performed using the GOLD 5.0 program. GoldScore was selected as the scoring function, and the most accurate genetic algorithm search option was set. A scaffold matching constraint was applied to position the benzene ring penetrating the protein interior close to its original location in the template. All other parameters remained at their default values. The binding affinity of the activator was evaluated based on the scoring values, and a reasonable binding mode was selected via cluster analysis.

### 3.2. Synthesis of Target Compounds

All reagents used in this study are of analytical purity. The mass spectrometry used for analyzing and verifying the structure of the compounds is Agilent 1290 Infinity-6538 UHD and Accurate-Mass QTOF/MS (Agilent, Santa Clara, CA, USA), and the proton nuclear magnetic resonance (^1^H NMR) and carbon nuclear magnetic resonance (^13^C NMR) spectra were obtained using a Bruker AV300/600 (Billerica, MA, USA). The purity was determined by an Agilent 1260 Infinity liquid chromatography system, with the chromatographic column being Agilent Eclipse Plus C18 (5 μm, 4.6 × 250mm). The purity of all the target compounds is >95%.

General method for step a: A solution of Halides (9.9 mmol), amines (9.9 mmol), and K_2_CO_3_ (18.8 mmol) in DMSO (15 mL) was stirred at 80 °C. Completion of the reaction was confirmed by TLC (Route Ι: DCM/MeOH system, and Route II: PE/EA system). The reaction mixture was cooled and added to water (50 mL), then extracted with EA, and the organic phase was dried over sodium sulfate anhydrous, filtered, and concentrated. The residue was partially purified by silica gel column (Route I: DCM/MeOH = 10:1, and Route II: PE/EA = 4:1) to afford the products with 79–99% yield.

General method for step b: Amines (1.1 mmol) were added in one portion at room temperature to a DCM solution (5 mL) of benzoic acids (1.0 mmol), EDCI (5.01 mmol), and DMAP (2.05 mmol). The resulting solution was allowed to stir at room temperature (1.5–6 h) under argon. The reaction was diluted with DCM, and washed with water (2×) and a saturated aq NaCl solution. The organic phase was dried over sodium sulfate anhydrous, filtered, and concentrated. The residue was purified by silica gel column (PE/EA = 3:1) to give the products with 67–95% yield.

General method for step c: Nitro compounds (0.06 mmol) were dissolved in EtOH (15 mL); NH_4_Cl (0.24 mmol) was dissolved in water (5 mL), and added to the reaction mixture; the system was refluxed at 80 °C for 0.5 h, and, then, iron powder (0.23 mmol) was added; and the reaction mixture and heated at 80 °C for 1–3 h. Completion of the reaction was confirmed by TLC. The reaction was cooled to room temperature, filtered over a plug of Celite, and the filtrate was combined and concentrated in vacuo. The residue was purified by silica gel column (PE/EA = 2:1) to afford the products with 71–97% yield.

General method for step d: Amines (0.84 mmol) and 3-fluoro-4-methoxybenzaldehyde (0.84 mmol) in DCE were stirred at 80 °C for ~1 h and cooled to room temperature, and additional sodium triacetoxyhydroborate (1.67 mmol) was added and allowed to react at room temperature under argon atmosphere. The reaction was quenched by saturated sodium bicarbonate solution, and the reaction mixture was extracted with DCM (50 mL) three times. The combined organics were dried over anhydrous Na_2_SO_4_, and concentrated by rotary evaporator, and then purified by silica gel column (PE/EA = 1:1) to provide the products with 59–95% yield.

General method for step e: Tert butyl carboxylates were dissolved in DCM, and additional TFA was added; and the reaction was stirred at room temperature for 0.5–2.5 h. Completion of the reaction was confirmed by TLC. The reaction mixture was concentrated under reduced pressure and the residue was partially purified by reversed-phase chromatography (C18 column, gradient elution with MeOH/H_2_O), and the product was obtained after freeze-drying in 74–95% yield.

Method for step f: Esters (1.0 mmol) were dissolved in the mixed solvent with a MeOH:THF ratio of 1:1 (8 mL), and NaOH (10.0 mmol) in 5:3 MeOH:H_2_O was added dropwise to the reaction mixture at room temperature. Completion of the reaction was confirmed by TLC. The reaction mixture was concentrated by rotary evaporator; the residue was extracted with 1M diluted HCl and DCM; the combined organic phase was concentrated by rotary evaporator; and the solid was separated with 91% yield.

Method for step g: Tert-butyl 4-(2-((cyclopropylmethyl)carbamoyl)-4-((3-fluoro-4-methoxybenzyl)amino)benzyl)piperazine-1-carboxylate (972 mg, 1.84 mmol) was dissolved in EA (10 mL); and concentrated HCl (0.5 mL) was added and stirred at room temperature for 1.5 h. The mixture was repeatedly dissolved with DCM, stirred, and concentrated to give a white solid (543 mg) in 69% yield.

Method for step h: Degassed DMF (6 mL) was added to 5-Nitroisobenzofuran-1,3-dione (300 mg, 1.6 mmol), tert-Butyl 4-aminopiperazine-1-carboxylate (311 mg, 1.6 mmol), HATU (2.36 g, 4.0 mmol), and DIPEA (1.08 mL, 4.0 mmol) under argon. The suspension was degassed by ultrasound for 30 min, refilled with argon, heated to 80 °C for 12 h, and then cooled to room temperature; the reaction mixture was extracted with diluted HCl and DCM; the combined organic phase was dried with anhydrous Na_2_SO_4_, concentrated by rotary evaporator; and the residue was purified by silica gel column (DCM:MeOH = 15:1) to afford a yellow solid (261.9 mg) with 42.8% yield.

Method for step i: 2-Bromo-*N*-(cyclopropylmethyl)-5-nitrobenzamide (50 mg, 0.17 mmol), tert-butyl 4-(4,4,5,5-tetramethyl-1,3,2-dioxaborolan-2-yl)-5,6-dihydropyridine-1(2H)-carboxylate (52 mg, 0.17 mmol), PdXPhosG_2_ (1.34 mg, 0.0017 mmol), Pd/C (0.02 mmol), and K_3_PO_4_ (107 mg, 0.50 mmol) were dissolved in 5 mL of a Dioxane: H_2_O mixed solvent with a ratio of 4:1 under argon atmosphere. The solution was heated to 80 °C. After 4 h, the reaction mixture was cooled to room temperature; and NH_4_COOH (106 mg, 1.68 mmol) was dissolved in MeOH (2 mL), poured into the reaction mixture, and reacted at room temperature for 16 h. Completion of the reaction was confirmed by TLC. The reaction was filtered through celite, concentrated by rotary evaporation, and purified by silica gel column (PE/EA = 1:1) to afford a white solid (47 mg) with 75% yield.

General method for step j: A degassed mixture of aryl bromides (0.17 mmol), boric acid pinacol esters (0.17 mmol), Pd(dppf)Cl_2_ (0.016 mmol), and K_2_CO_3_ (0.5 mmol) in a Dioxane:H_2_O mixed solvent with a ratio of 3:1 was heated at 100 °C for 12 h. The reaction mixture was diluted with EA and washed with water. The aqueous layer was extracted with EA. The combined organics were washed with brine, dried (Na_2_SO_4_), and concentrated. The residue was purified by silica gel column (PE/EA = 2:1) to provide a white solid with ~70% yield.

*N*-((1-aminocyclopropyl)methyl)-5-((3-fluoro-4-methoxybenzyl)amino)-2-morpholinobenzamide (**D1**): Brown solid, ^1^H NMR (600 MHz, DMSO-*d*_6_) δ 10.52 (t, *J* = 5.9 Hz, 1H), 8.40–8.28 (m, 3H), 7.24 (d, *J* = 2.9 Hz, 1H), 7.18–7.13 (m, 2H), 7.12–7.07 (m, 2H), 6.68 (dd, *J* = 8.7, 3.0 Hz, 1H), 4.20 (s, 2H), 3.78 (s, 3H), 3.75–3.70 (m, 4H), 3.57 (d, *J* = 6.1 Hz, 2H), 2.85–2.79 (m, 4H), 0.94–0.90 (m, 2H), 0.90–0.86 (m, 2H). ^13^C NMR (151 MHz, DMSO) δ 167.04, 151.44 (d, *J* = 243.8 Hz), 145.76 (d, *J* = 10.4 Hz), 145.70, 140.01, 133.12 (d, *J* = 4.3 Hz), 128.48, 123.05, 122.79, 115.36, 114.48 (d, *J* = 17.9 Hz), 113.82, 113.77, 66.65, 55.99, 53.30, 45.53, 42.84, 34.85, 8.96. HRMS (ESI): *m*/*z* calcd for C_23_H_29_FN_4_O_3_ (M + H)^+^: 429.2224, found 429.2326.

*N*-(2-amino-2-methylpropyl)-5-((3-fluoro-4-methoxybenzyl)amino)-2-morpholinobenzamide (**D2**): Yellow solid, ^1^H NMR (600 MHz, DMSO-*d*_6_) δ 10.61 (t, 1H), 7.86 (s, 3H), 7.25 (d, *J* = 3.0 Hz, 1H), 7.21 (d, *J* = 8.7 Hz, 1H), 7.15 (d, 1H), 7.12–7.07 (m, 2H), 6.70 (dd, *J* = 8.7, 3.0 Hz, 1H), 4.21 (s, 2H), 3.79 (s, 3H), 3.74–3.71 (m, 4H), 3.49 (d, *J* = 6.6 Hz, 2H), 2.87–2.80 (m, 4H), 1.26 (s, 6H). ^13^C NMR (151 MHz, DMSO) δ 167.16, 151.42 (d, *J* = 243.6 Hz), 145.77 (d, *J* = 3.9 Hz), 145.72, 139.86, 133.09 (d, *J* = 5.0 Hz), 128.51, 123.05, 122.98, 115.31, 114.47 (d, *J* = 17.9 Hz), 113.85, 113.76, 66.62, 55.98, 54.55, 53.31, 46.27, 45.52, 23.40. HRMS (ESI): *m*/*z* calcd for C_23_H_31_FN_4_O_3_ (M + H)^+^: 431.2477, found 431.2380.

*N*-(cyclopropylmethyl)-5-((3-fluoro-4-methoxybenzyl)amino)-2-(piperazin-1-yl)benzamide (**D5**): White solid, ^1^H NMR (300 MHz, Chloroform-*d*) δ 10.72 (t, *J* = 5.1 Hz, 1H), 7.57 (d, *J* = 3.0 Hz, 1H), 7.17–6.99 (m, 3H), 6.90 (t, *J* = 8.6 Hz, 1H), 6.62 (dd, *J* = 8.6, 3.0 Hz, 1H), 4.27 (s, 2H), 3.86 (s, 3H), 3.30 (dd, *J* = 7.1, 5.1 Hz, 2H), 3.07 (t, *J* = 4.7 Hz, 4H), 2.90 (t, *J* = 4.7 Hz, 4H), 2.48 (s, 1H), 1.06 (ddd, *J* = 12.4, 7.8, 4.7 Hz, 1H), 0.56 (dt, *J* = 8.4, 2.9 Hz, 2H), 0.29 (t, *J* = 5.0 Hz, 2H). ^13^C NMR (101 MHz, DMSO-*d*_6_) δ 165.62, 151.44 (d, *J* = 243.6 Hz), 145.73 (d, *J* = 11.1 Hz), 145.63, 139.55, 133.18 (d, *J* = 5.4 Hz), 129.39, 123.03 (d, *J* = 3.3 Hz), 122.01, 114.60, 114.47 (d, *J* = 19.2 Hz), 113.97, 113.75, 55.96, 51.37, 45.52, 44.41, 43.44, 10.86, 3.44. HRMS (ESI): *m*/*z* calcd for C_23_H_29_FN_4_O_2_ (M + H)^+^: 413.2275, found 413.44.

N-(cyclopropylmethyl)-5-((3-fluoro-4-methoxybenzyl)amino)-2-(piperazin-1-ylmethyl)benzamide (**D10**): White solid, ^1^H NMR (600 MHz, DMSO-*d*_6_) δ 9.97 (t, *J* = 5.2 Hz, 1H), 7.21–7.14 (m, 1H), 7.13–7.09 (m, 2H), 6.99–6.91 (m, 2H), 6.56 (dd, *J* = 8.3, 2.7 Hz, 1H), 6.42 (t, *J* = 6.1 Hz, 1H), 4.21 (d, *J* = 6.1 Hz, 2H), 3.81 (s, 3H), 3.43 (s, 2H), 3.13 (dd, *J* = 6.8, 5.3 Hz, 2H), 2.66 (s, 4H), 2.30 (s, 4H), 1.04–0.93 (m, 2H), 0.53–0.42 (m, 2H), 0.25–0.15 (m, 2H). ^13^C NMR (101 MHz, DMSO-*d*_6_) δ 168.26, 151.43 (d, *J* = 243.6 Hz), 147.83, 145.78 (d, *J* = 10.6 Hz), 138.09, 133.12 (d, *J* = 5.3 Hz), 132.32, 123.10 (d, *J* = 3.6 Hz), 120.37, 114.52 (d, *J* = 18.0 Hz), 113.75, 113.11, 112.51, 59.60, 55.97, 49.29, 45.34, 43.62, 43.32, 10.82, 3.36. HRMS (ESI): *m*/*z* calcd for C_24_H_31_FN_4_O_2_ (M + H)^+^: 427.2431, found 427.2554.

*N*-(cyclopropylmethyl)-5-((3-fluoro-4-methoxybenzyl)amino)-2-(piperidin-1-ylmethyl)benzamide (**D11**): White solid, ^1^H NMR (300 MHz, Chloroform-*d*) δ 9.41 (s, 1H), 7.17–7.02 (m, 4H), 6.89 (t, *J* = 8.6 Hz, 1H), 6.50 (dd, *J* = 8.2, 2.5 Hz, 1H), 4.25 (s, 2H), 3.85 (s, 3H), 3.66 (s, 2H), 3.23 (dd, *J* = 6.9, 5.1 Hz, 2H), 2.74 (s, 4H), 2.01 (s, 2H), 1.69–1.49 (m, 6H), 1.07 (qq, *J* = 7.4, 4.8, 3.6 Hz, 1H), 0.57–0.45 (m, 2H), 0.31–0.20 (m, 2H). ^13^C NMR (151 MHz, Chloroform-*d*) δ 170.09, 152.51 (d, *J* = 246.2 Hz), 149.04, 146.89 (d, *J* = 10.5 Hz), 137.48, 134.56, 131.53, 123.12, 115.61, 115.10 (d, *J* = 18.4 Hz), 114.33, 113.69, 113.26, 56.31 (d, *J* = 16.2 Hz), 52.01, 45.07 (d, *J* = 30.1 Hz), 23.27 (d, *J* = 38.7 Hz), 21.97 (d, *J* = 19.0 Hz), 10.67, 3.66 (d, *J* = 11.2 Hz). HRMS (ESI): *m*/*z* calcd for C_25_H_32_FN_3_O_2_ (M + H)^+^: 426.2479, found 426.2555.

*N*-(cyclopropylmethyl)-2-((dimethylamino)methyl)-5-((3-fluoro-4-methoxybenzyl)amino)benzamide (**D16**): Gray solid, ^1^H NMR (600 MHz, Chloroform-*d*) δ 8.45 (s, 1H), 7.29 (s, 2H), 7.07 (td, *J* = 7.5, 3.8 Hz, 3H), 6.91 (t, *J* = 8.6 Hz, 1H), 6.55 (dd, *J* = 8.3, 2.5 Hz, 1H), 4.28 (s, 2H), 3.97 (s, 2H), 3.87 (s, 3H), 3.23 (dd, *J* = 7.5, 4.1 Hz, 2H), 2.68 (s, 6H), 1.09 (s, 1H), 0.56–0.49 (m, 2H), 0.28 (dt, *J* = 6.1, 4.6 Hz, 2H). ^13^C NMR (151 MHz, Chloroform-*d*) δ 153.33, 151.69, 149.57, 146.89 (d, *J* = 10.9 Hz), 136.90, 134.14, 131.39, 122.95 (d, *J* = 3.6 Hz), 115.71, 115.06, 114.94, 113.95, 113.70, 113.48, 60.04, 56.34, 46.73, 45.15, 44.58, 41.78, 22.41 (d, *J* = 24.9 Hz), 10.40, 3.49. HRMS (ESI): *m*/*z* calcd for C_22_H_28_FN_3_O_2_ (M + H)^+^: 386.2166, found 386.2256.

*N*-(cyclopropylmethyl)-5-((3-fluoro-4-methoxybenzyl)amino)-2-(pyridin-3-yl)benzamide (**D20**): White solid, ^1^H NMR (600 MHz, Chloroform-*d*) δ 8.62 (d, *J* = 2.3 Hz, 1H), 8.52 (dd, *J* = 4.9, 1.6 Hz, 1H), 7.71 (dt, *J* = 7.9, 2.0 Hz, 1H), 7.30–7.28 (m, 1H), 7.17 (d, *J* = 8.3 Hz, 1H), 7.12–7.04 (m, 2H), 6.96–6.90 (m, 2H), 6.71 (dd, *J* = 8.4, 2.6 Hz, 1H), 5.45 (t, *J* = 5.4 Hz, 1H), 4.33 (s, 2H), 3.89 (s, 3H), 3.03 (dd, *J* = 7.2, 5.5 Hz, 2H), 0.73–0.58 (m, 1H), 0.38–0.27 (m, 2H), −0.00–−0.04 (m, 2H). HRMS (ESI): *m*/*z* calcd for C_24_H_24_FN_3_O_2_ (M + H)^+^: 406.1853, found 406.1921.

5-((3-fluoro-4-methoxybenzyl)amino)-*N*-isopentyl-2-(piperazin-1-yl)benzamide (**D25**): White solid, ^1^H NMR (600 MHz, DMSO-*d*_6_) δ 10.44 (s, 1H), 7.23 (d, *J* = 3.0 Hz, 1H), 7.15 (d, *J* = 12.4 Hz, 1H), 7.09 (d, *J* = 16.8 Hz, 3H), 6.63 (d, *J* = 8.7 Hz, 1H), 6.32 (s, 1H), 4.18 (d, *J* = 6.3 Hz, 2H), 3.79 (s, 3H), 2.81 (s, 4H), 2.69 (s, 4H), 2.61 (s, 1H), 2.38 (s, 1H), 1.66–1.60 (m, 1H), 1.43 (d, *J* = 7.3 Hz, 2H), 1.23 (s, 2H), 0.91(d, *J* = 6.6 Hz, 5H). ^13^C NMR (151 MHz, Chloroform-*d*) δ 166.16 (d, *J* = 11.9 Hz), 153.34, 151.71, 145.81, 140.69, 140.10, 132.18, 129.02, 122.97 (d, *J* = 3.4 Hz), 115.58, 115.39 (d, *J* = 11.6 Hz), 115.19, 115.07, 113.59, 56.35, 53.28, 51.83, 47.45 (d, *J* = 43.3 Hz), 45.15, 38.81 (d, *J* = 4.2 Hz), 37.86 (d, *J* = 11.2 Hz), 26.17, 22.53. HRMS (ESI): *m*/*z* calcd for C_24_H_33_FN_4_O_2_ (M + H)^+^: 429.2588, found 429.2675.

*N*-(2-(dimethylamino)-2-methylpropyl)-5-((3-fluoro-4-methoxybenzyl)amino)-2-morpholinobenzamide (**D3**): White solid, ^1^H NMR (600 MHz, DMSO-*d*_6_) δ 10.75 (t, *J* = 6.8 Hz, 1H), 7.27 (d, *J* = 3.0 Hz, 1H), 7.22 (d, *J* = 8.7 Hz, 1H), 7.17–7.13 (m, 1H), 7.12–7.07 (m, 2H), 6.70 (dd, *J* = 8.7, 3.0 Hz, 1H), 6.45 (t, *J* = 6.2 Hz, 1H), 4.22 (d, *J* = 6.1 Hz, 2H), 3.79 (s, 3H), 3.75–3.70 (m, 4H), 3.67 (d, *J* = 7.4 Hz, 2H), 2.85–2.80 (m, 4H), 2.77 (s, 6H), 1.33 (s, 6H). HRMS (ESI): *m*/*z* calcd for C_25_H_35_FN_4_O_3_ (M + H)^+^: 459.2693, found 459.2273.

*N*-(2-aminoethyl)-5-((3-fluoro-4-methoxybenzyl)amino)-2-morpholinobenzamide (**D4**): White solid, ^1^H NMR (600 MHz, DMSO-*d*_6_) δ 10.17–10.01 (m, 1H), 7.21–7.13 (m, 2H), 7.11–7.03 (m, 3H), 6.62 (td, *J* = 8.6, 3.0 Hz, 1H), 6.33 (dt, *J* = 13.0, 6.2 Hz, 1H), 4.18 (dd, *J* = 6.2, 2.5 Hz, 2H), 3.79 (s, 3H), 3.73 (dt, *J* = 21.5, 4.7 Hz, 4H), 3.28 (dd, *J* = 10.5, 5.6 Hz, 3H), 3.07 (q, *J* = 6.0 Hz, 1H), 2.81–2.73 (m, 4H), 2.70 (t, *J* = 6.2 Hz, 1H). HRMS (ESI): *m*/*z* calcd for C_21_H_27_FN_4_O_3_ (M + H)^+^: 403.2067, found 403.2174.

*N*-(cyclopropylmethyl)-5-((3-fluoro-4-methoxybenzyl)amino)-2-(4-methylpiperazin-1-yl)benzamide (**D6**): White solid, ^1^H NMR (300 MHz, Chloroform-*d*) δ 10.53 (s, 1H), 7.56 (d, *J* = 2.8 Hz, 1H), 7.17–7.01 (m, 3H), 6.97–6.84 (m, 1H), 6.69–06.56 (m, 1H), 4.28 (s, 2H), 3.87 (s, 3H), 3.31 (t, *J* = 6.2 Hz, 2H), 3.11–2.92 (m, 4H), 2.70 (s, 4H), 2.42 (s, 3H), 1.14–0.98 (m, 1H), 0.67–0.50 (m, 2H), 0.36–0.21 (m, 2H). HRMS (ESI): *m*/*z* calcd for C_24_H_31_FN_4_O_2_ (M + H)^+^: 427.2631, found 427.2506.

*N*-(cyclopropylmethyl)-2-(4-ethylpiperazin-1-yl)-5-((3-fluoro-4-methoxybenzyl)amino)benzamide (**D7**): White solid, ^1^H NMR (600 MHz, Chloroform-*d*) δ 10.71 (s, 1H), 7.58 (d, *J* = 3.0 Hz, 1H), 7.14 (d, *J* = 8.6 Hz, 1H), 7.10–7.05 (m, 2H), 6.92 (t, *J* = 8.4 Hz, 1H), 6.64 (dd, *J* = 8.6, 3.0 Hz, 1H), 4.29 (s, 2H), 3.88 (s, 3H), 3.32 (dd, *J* = 7.1, 5.1 Hz, 2H), 3.00 (t, *J* = 4.9 Hz, 4H), 2.68 (s, 4H), 2.52 (q, *J* = 7.2 Hz, 2H), 1.16 (t, *J* = 7.2 Hz, 3H), 1.11–1.02 (m, 1H), 0.63–0.54 (m, 2H), 0.34–0.25 (m, 2H). HRMS (ESI): *m*/*z* calcd for C_25_H_33_FN_4_O_2_ (M + H)^+^: 441.2588, found 441.2677.

2-(4-aminopiperidin-1-yl)-*N*-(cyclopropylmethyl)-5-((3-fluoro-4-methoxybenzyl)amino)benzamide (**D8**): White solid, ^1^H NMR (300 MHz, Methanol-*d*_4_) δ 7.48 (dd, *J* = 9.2, 4.1 Hz, 1H), 7.25 (d, *J* = 3.4 Hz, 1H), 7.11 (d, *J* = 12.9 Hz, 3H), 6.91 (dt, *J* = 8.4, 3.4 Hz, 1H), 4.45–4.28 (m, 2H), 3.93–3.79 (m, 3H), 3.53 (s, 5H), 2.37 (d, *J* = 13.5 Hz, 2H), 2.03 (d, *J* = 11.9 Hz, 2H), 1.15 (s, 1H), 0.59 (t, *J* = 5.7 Hz, 2H), 0.33 (d, *J* = 4.6 Hz, 2H). HRMS (ESI): *m*/*z* calcd for C_24_H_31_FN_4_O_2_ (M + H)^+^: 427.2431, found 427.2508.

2-((2-aminoethyl)(methyl)amino)-*N*-(cyclopropylmethyl)-5-((3-fluoro-4-methoxybenzyl)amino)benzamide (**D9**): Gray solid, ^1^H NMR (600 MHz, DMSO-*d*_6_) δ 9.46 (s, 1H), 7.19–7.09 (m, 1H), 6.82 (d, *J* = 2.9 Hz, 4H), 6.64 (dd, *J* = 8.7, 2.9 Hz, 1H), 6.38 (t, *J* = 6.1 Hz, 1H), 4.19 (d, *J* = 6.1 Hz, 2H), 3.80 (s, 3H), 3.16–3.10 (m, 2H), 2.99 (t, *J* = 6.1 Hz, 2H), 2.68 (t, *J* = 6.1 Hz, 2H), 2.53 (s, 3H), 1.04–0.96 (m, 1H), 0.47–0.38 (m, 2H), 0.24–0.18 (m, 2H). HRMS (ESI): *m*/*z* calcd for C_22_H_29_FN_4_O_2_ (M + H)^+^: 401.2275, found 401.2386.

5-((3-fluoro-4-methoxybenzyl)amino)-*N*-((1-hydroxycyclopropyl)methyl)-2-(piperazin-1-yl)benzamide (**D23**): White solid, ^1^H NMR (600 MHz, DMSO-*d*_6_) δ 10.10 (t, *J* = 5.1 Hz, 1H), 8.98 (s, 2H), 7.23 (d, *J* = 2.9 Hz, 1H), 7.14 (d, 1H), 7.11–7.05 (m, 3H), 6.66 (dd, *J* = 8.7, 3.0 Hz, 1H), 4.19 (s, 2H), 3.78 (s, 3H), 3.36 (d, *J* = 5.0 Hz, 2H), 3.31–3.27 (m, 4H), 3.02–2.97 (m, 4H), 1.22 (s, 1H), 0.66–0.62 (m, 2H), 0.56–0.52 (m, 2H). HRMS (ESI): *m*/*z* calcd for C_23_H_29_FN_4_O_3_ (M + H)^+^: 429.2224, found 429.2313.

5-((3-fluoro-4-methoxybenzyl)amino)-*N*-(2-hydroxy-2-methylpropyl)-2-(piperazin-1-yl)benzamide (**D24**): White solid, ^1^H NMR (600 MHz, DMSO-*d*_6_) δ 10.13 (t, *J* = 5.5 Hz, 1H), 8.90 (s, 2H), 7.28 (d, *J* = 3.0 Hz, 1H), 7.16 (d, *J* = 12.8 Hz, 1H), 7.12–7.07 (m, 3H), 6.67 (dd, *J* = 8.7, 3.0 Hz, 1H), 4.20 (s, 2H), 3.79 (s, 3H), 3.34–3.28 (m, 4H), 3.27 (d, *J* = 5.5 Hz, 2H), 3.02–2.96 (m, 4H), 1.13 (s, 6H). HRMS (ESI): *m*/*z* calcd for C_23_H_31_FN_4_O_3_ (M + H)^+^: 431.2380, found 431.2478.

*N*-(cyclopropylmethyl)-5-((3-fluoro-4-methoxybenzyl)amino)-*N*-methyl-2-(piperazin-1-yl)benzamide (**D26**): White solid, ^1^H NMR (600 MHz, DMSO-*d*_6_) δ 7.19–7.06 (m, 3H), 6.94–6.85 (dd, *J* = 8.7, 22.6 Hz, 1H), 6.59–6.53 (ddd, *J* = 2.8, 8.7, 14.9 Hz, 1H), 6.36–6.20 (m, 2H), 4.21–4.13 (t, *J* = 5.9 Hz, 2H), 3.83–3.76 (d, *J* = 8.3 Hz, 3H), 3.14–2.95 (m, 8H), 2.85–2.69 (m, 4H), 1.09–1.01 (s, 1H), 0.69–0.21 (m, 4H), 0.06–0.22 (m, 2H). HRMS (ESI): *m*/*z* calcd for C_24_H_31_FN_4_O_2_ (M + H)^+^: 427.2431, found 427.2514.

*N*-(cyclopropylmethyl)-5-((3-fluoro-4-methoxybenzyl)amino)-2-(pyrrolidin-1-ylmethyl)benzamide (**D12**): White solid, ^1^H NMR (600 MHz, Chloroform-*d*) δ 8.91 (s, 1H), 7.28 (d, *J* = 8.3 Hz, 1H), 7.09–7.00 (m, 3H), 6.91 (t, *J* = 8.6 Hz, 1H), 6.57 (dd, *J* = 8.3, 2.6 Hz, 1H), 4.26 (s, 2H), 3.86 (s, 3H), 3.24 (dd, *J* = 7.1, 4.5 Hz, 2H), 2.89 (d, *J* = 6.7 Hz, 4H), 2.03 (s, 2H), 1.92 (p, *J* = 3.3 Hz, 4H), 1.06–0.99 (m, 1H), 0.55–0.49 (m, 2H), 0.25 (dd, *J* = 4.7, 1.3 Hz, 2H). HRMS (ESI): *m*/*z* calcd for C_24_H_30_FN_3_O_2_ (M + H)^+^: 412.2322, found 412.2410.

2-(((2-aminoethyl)(methyl)amino)methyl)-*N*-(cyclopropylmethyl)-5-((3-fluoro-4-methoxybenzyl)amino)benzamide (**D14**): Light yellow solid powder, ^1^H NMR (600 MHz, DMSO-*d*_6_) δ 9.37–9.18 (m, 1H), 9.01–8.90 (d, *J* = 7.0 Hz, 1H), 8.28–8.12 (s, 2H), 7.28–7.07 (m, 4H), 6.95–6.85 (s, 1H), 6.73–6.64 (d, *J* = 8.3 Hz, 1H), 4.34–4.24 (s, 2H), 4.11–3.97 (s, 1H), 3.83 –3.77 (s, 3H), 3.30–3.06 (m, 7H), 2.73–2.58 (s, 3H), 1.11–0.95 (s, 1H), 0.52–0.38 (d, *J* = 7.7 Hz, 2H), 0.29–0.16 (d, *J* = 5.0 Hz, 2H). HRMS (ESI): *m*/*z* calcd for C_23_H_31_FN_4_O_2_ (M + H)^+^: 415.2431, found 415.2519.

2-(((3-aminopropyl)(methyl)amino)methyl)-*N*-(cyclopropylmethyl)-5-((3-fluoro-4-methoxybenzyl)amino)benzamide (**D15**): White solid, ^1^H NMR (600 MHz, DMSO-*d*_6_) δ 9.21 (s, 1H), 8.04–7.65 (m, 3H), 7.20–7.00 (m, 4H), 6.85 (d, *J* = 2.5 Hz, 1H), 6.60 (s, 1H), 4.23 (d, *J* = 5.9 Hz, 2H), 3.80 (s, 3H), 3.36 (s, 4H), 3.12 (t, *J* = 6.2 Hz, 2H), 2.76 (d, *J* = 20.6 Hz, 2H), 2.41–2.08 (m, 3H), 1.78 (s, 2H), 1.00 (s, 1H), 0.49–0.42 (m, 2H), 0.21 (dt, *J* = 6.1, 2.9 Hz, 2H). HRMS (ESI): *m*/*z* calcd for C_24_H_33_FN_4_O_2_ (M + H)^+^: 429.2588, found 429.2674.

*N^2^*-(cyclopropylmethyl)-4-((3-fluoro-4-methoxybenzyl)amino)-*N^1^*-(piperidin-4-yl)phthalamide (**D17**): ^1^H NMR (600 MHz, DMSO-*d*_6_) δ 8.26–8.22 (m, 1H), 8.04–7.94 (m, 1H), 7.30 (dd, *J* = 32.7, 8.4 Hz, 1H), 7.18–7.07 (m, 3H), 6.79 (dt, *J* = 12.5, 6.2 Hz, 1H), 6.62–6.51 (m, 2H), 4.26 (d, *J* = 6.2 Hz, 2H), 3.95–3.87 (m, 1H), 3.80 (d, *J* = 1.2 Hz, 3H), 3.28–3.20 (m, 2H), 3.11–2.94 (m, 4H), 2.01–1.85 (m, 2H), 1.68–1.51 (m, 2H), 1.00–0.89 (m, 1H), 0.39 (dddd, *J* = 13.8, 8.2, 5.9, 4.1 Hz, 2H), 0.25–0.14 (m, 2H). HRMS (ESI): *m*/*z* calcd for C_25_H_31_FN_4_O_3_ (M + H)^+^: 455.2380, found 455.2470.

*N*-(cyclopropylmethyl)-5-((3-fluoro-4-methoxybenzyl)amino)-2-(pyridin-4-yl)benzamide (**D19**): White solid, ^1^H NMR (400 MHz, Chloroform-*d*) δ 8.77–8.24 (m, 2H), 7.36–7.29 (m, 2H), 7.20 (dd, *J* = 8.7, 2.1 Hz, 1H), 7.08 (m, 2H), 6.94 (t, *J* = 8.0 Hz, 1H), 6.88 (d, *J* = 2.9 Hz, 1H), 6.75–6.66 (m, 1H), 5.42 (d, *J* = 5.8 Hz, 1H), 4.33 (s, 2H), 3.89 (s, 3H), 3.04 (t, *J* = 6.5 Hz, 2H), 0.75–0.56 (m, 1H), 0.40–0.28 (m, 2H), 0.04–−0.10 (m, 2H). HRMS (ESI): *m*/*z* calcd for C_24_H_24_FN_3_O_2_ (M + H)^+^: 406.1853, found 406.1934.

*N*-(cyclopropylmethyl)-5-((3-fluoro-4-methoxybenzyl)amino)-2-(piperidin-4-yl)benzamide (**D18**): White solid, ^1^H NMR (600 MHz, Methanol-*d*_4_) δ 7.14–7.06 (m, 3H), 7.01 (t, *J* = 8.6 Hz, 1H), 6.74 (dd, *J* = 8.6, 2.6 Hz, 1H), 6.67 (d, *J* = 2.5 Hz, 1H), 4.29 (s, 2H), 3.83 (s, 3H), 3.49–3.39 (m, 2H), 3.19 (d, *J* = 7.0 Hz, 2H), 3.06–2.97 (m, 3H), 2.02 (d, *J* = 14.1 Hz, 2H), 1.91–1.77 (m, 2H), 1.13–1.00 (m, 1H), 0.58–0.44 (m, 2H), 0.28–0.22 (m, 2H). HRMS (ESI): *m*/*z* calcd for C_24_H_30_FN_3_O_2_ (M + H)^+^: 412.2322, found 412.2396.

*N*-(cyclopropylmethyl)-5-((3-fluoro-4-methoxybenzyl)amino)-2-(5-hydroxypyridin-3-yl)benzamide (**D21**): White solid, ^1^H NMR (300 MHz, Chloroform-*d*) δ 8.16 (dd, *J* = 11.6, 2.2 Hz, 1H), 7.41 (t, *J* = 2.1 Hz, 1H), 7.28 (s, 1H), 7.20 (t, *J* = 7.8 Hz, 1H), 7.12–6.97 (m, 4H), 6.91 (t, *J* = 8.6 Hz, 1H), 6.71 (dd, *J* = 8.1, 2.4 Hz, 1H), 6.37 (s, 1H), 4.28 (s, 1H), 3.87 (s, 3H), 3.29 (dd, *J* = 7.2, 5.3 Hz, 2H), 1.17–0.95 (m, 1H), 0.62–0.49 (m, 2H), 0.37–0.18 (m, 2H). HRMS (ESI): *m*/*z* calcd for C_24_H_24_FN_3_O_3_ (M + H)^+^: 422.1802, found 422.16.

*N*-(cyclopropylmethyl)-5-((3-fluoro-4-methoxybenzyl)amino)-2-(1H-pyrazol-4-yl)benzamide (**D22**): White solid, ^1^H NMR (600 MHz, Chloroform-*d*) δ 7.62 (s, 2H), 7.51 (d, *J* = 7.5 Hz, 1H), 7.12–7.04 (m, 2H), 7.01–6.91 (m, 3H), 6.46 (s, 1H), 4.66 (s, 1H), 4.32 (s, 2H), 3.84 (s, 3H), 3.32 (d, *J* = 7.0 Hz, 2H), 1.01 (hept, *J* = 7.0 Hz, 1H), 0.45 (ddd, *J* = 7.1, 6.0, 4.2 Hz, 2H), 0.28–0.20 (m, 2H). HRMS (ESI): *m*/*z* calcd for C_22_H_23_FN_4_O_2_ (M − H)^−^: 393.1805, found 393.44.

### 3.3. Determination of the Solubility of Target Compounds

Precisely weigh the compound, add a certain volume of solvent to dissolve the compound until a clear solution is obtained, and prepare a control solution with a known concentration. Add a certain volume of pure water to the sample and dissolve it until saturated. The clear solution obtained after filtration is the solution to be tested. Use an Agilent 1260 Infinity liquid chromatography system with an Agilent Eclipse Plus C18 (5 μm, 4.6 × 250mm) chromatographic column, and perform gradient elution with methanol/water to measure the control solution and the solution to be tested. Calculate the concentration of the compound in the solution to be tested through the formula: *C*_(solution to be tested)_/*C*_(control solution)_ = *A*_(solution to be tested)_/*A*_(control solution)_, where *C* is the compound concentration and *A* is the peak area of the compound. The concentration of the compound in the solution to be tested corresponds to its solubility in pure water.

### 3.4. Evaluation of the ALDH2 Activation Activity of Target Compounds

The test employed the ALDH2 test kit (Abcam ab115348, Cambridge, UK) and the active human ALDH2 protein (Abcam ab87415). The testing principle is that ALDH2 catalyzes the conversion of acetaldehyde into acetic acid, with the concomitant reduction in the coenzyme NAD^+^ to NADH. The generated NADH reacts with a dye and has an absorption at 450 nm. The slope of the absorbance–time curve, fitted using linear regression, serves as the metric for enzyme activity. During the test, the concentration of ALDH2 protein was fixed at 5 μg/mL, and the procedures followed the instruction manual of the kit. The maximum activation fold was calculated as the activity of ALDH2 in the presence of test compounds at 100 μM, which is calibrated against Alda-1. The calculation formula is as follows: maximum activation fold = (compound − min)/(max − min) × 100%, where “compound”: slope of the absorbance–time curve for the target compound group; “min”: slope of the absorbance time curve for the blank control group (DMSO); “max”: the slope of the absorbance time curve for the positive control group (Alda-1).

### 3.5. Evaluation of the Protective Effect of Target Compounds on Animal Models of Cerebral Infarction

All operations complied with the ethical requirements for laboratory animals of the Naval Medical University. Male Sprague–Dawley (SD) rats (240–270 g) were selected, weighed, numbered, and randomly grouped. They were fasted for 12 h before the operation but had free access to water. The model was established using the middle cerebral artery occlusion (MCAO) method. After successful induction of ischemia for 15 min via MCAO surgery, samples were injected into the tail veins of the grouped rats. Then, after 2 h of ischemia, reperfusion was performed for 24 h. Subsequently, the rats were anesthetized by intraperitoneal injection of 2% pentobarbital at a dose of 50 mg/kg. The chest cavity was opened to expose the heart, and the heart was perfused with normal saline. Paleness of the rats’ upper limbs and head, along with clear outflowing liquid, indicated successful perfusion. The rat’s head was then decapitated, and the brain tissue was carefully extracted and immediately frozen at −20 °C for 20 min. The brain was then cut into 6 uniform-thickness coronal slices front to back. The cut coronal brain slices were placed in a 2% 2,3,5-triphenyltetrazolium chloride (TTC) staining solution for staining for 20 min at room temperature. The slices were then flipped with tweezers and stained for another 20 min at room temperature. Next, 4% paraformaldehyde was added for fixation for 6 h to preserve the brain tissue slices and prevent decay. The slices were carefully flattened on a transparent plastic film, and brain tissue slices from each group of rats were scanned and photographed using a scanner, and the percentage of the total infarcted area of the brain slices of the affected hemisphere was calculated.

## 4. Conclusions

In this study, aiming to address the existing limitations of the current ALDH2 activators, a systematic structural optimization was conducted based on the binding mode of *N*-benzylaniline derivatives and ALDH2. The study found that activity can only be maintained or improved by introducing amino groups or other basic groups at specific positions of the lead compound. Among these compounds, compound **D10** exhibited the optimal activity, with a maximum activation fold reached at 114% relative to Alda-1, and its EC_50_ value was 16.8 µM. Its hydrochloride salt **D27** showed a more than 200-fold increase in water solubility. The intravenous injection of this compound significantly reduced the infarct area in a rat model of cerebral infarction compared to the model group. However, this class of ALDH2 activator still requires further structural modification to enhance in vivo biological activity, and their pharmacokinetic properties also need further evaluation. Nevertheless, this study provides a solid foundation for the future research on ALDH2 activators used in the treatment of stroke.

## Figures and Tables

**Table 1:** Structural optimization of triazole ALDH2 activators.

No.	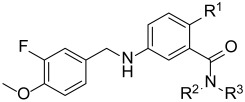			Maximum Activation Fold Calibrated Against Alda-1 (%) ^1^
	R^1^	R^2^	R^3^	
**C6**	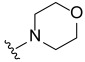	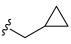	-H	104 ± 2
**D1**	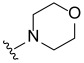	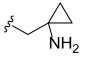	-H	99 ± 2
**D2**	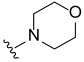	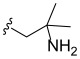	-H	103 ± 6
**D3**	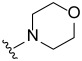	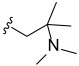	-H	65 ± 1
**D4**	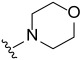	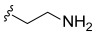	-H	13 ± 4
**D5**	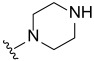	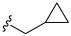	-H	103 ± 4
**D6**	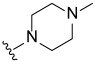	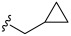	-H	32 ± 3
**D7**	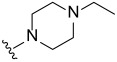	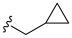	-H	67 ± 10
**D8**	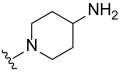	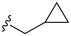	-H	38 ± 5
**D9**	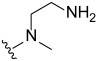	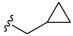	-H	110 ± 3
**D10**	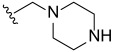	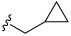	-H	118 ± 6
**D11**	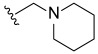	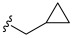	-H	99 ± 5
**D12**	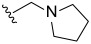	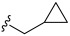	-H	87 ± 1
**D13**	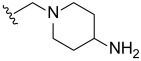	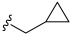	-H	59 ± 5
**D14**	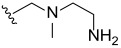	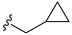	-H	13 ± 4
**D15**	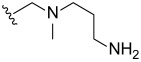	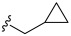	-H	36 ± 5
**D16**	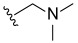	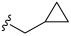	-H	65 ± 2
**D17**	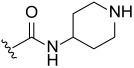	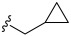	-H	47 ± 7
**D18**	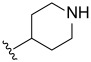	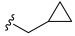	-H	22 ± 2
**D19**	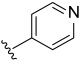	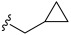	-H	55 ± 1
**D20**	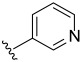	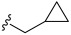	-H	71 ± 3
**D21**	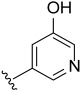	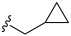	-H	86 ± 2
**D22**	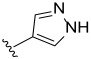	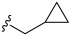	-H	73 ± 6
**D23**	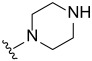	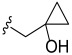	-H	85 ± 1
**D24**	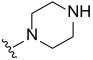	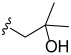	-H	99 ± 3
**D25**	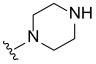	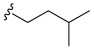	-H	100 ± 5
**D26**	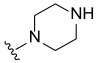	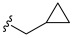	-CH_3_	92 ± 3

^1^ The maximum fold activation of Alda-1 was 1.98–2.44 in different tests, which was assigned as 100%.

## Data Availability

The original contributions presented in this study are included in the Supplementary Material. Further inquiries can be directed to the corresponding author.

## Supplementary Materials

The following supporting information can be downloaded at: https://www.mdpi.com/article/10.3390/molecules30142924/s1, ^1^H NMR, ^13^C NMR spectra of target compounds, and summary of the interactions between **C6** and **D10** and ALDH2.
