# Supplementary material for: Design, Synthesis, and Study of Protective Activity Against Stroke for Novel Water-Soluble Aldehyde Dehydrogenase 2 Activators
Source: Molecules. 2025 Jul 10;30(14):2924. doi: 10.3390/molecules30142924 (PMC12301025; doi:10.3390/molecules30142924)
Supplement: Supplementary file 1 [file molecules-30-02924-s001.zip › molecules-3638709-supplementary.pdf]

Supplementary Materials

Design, Synthesis, and Study of Protective Activity Against Stroke for Novel Water-Soluble Aldehyde Dehydrogenase 2 Activators

Fengping Zhao [1,2], Zhenming Yu [1,2], Wei Tian [3], Xinhui Huang [2], Qingsen Zhang [2], Ruolan Zhou [1,2], Jian Hu [2], Shichong Yu [2,*], Xin Chen [1,*] and Canhui Zheng [2,*]

[1] School of Life Science and Technology, Wuhan Polytechnic University, Wuhan 430023, China
[2] The Center for Basic Research and Innovation of Medicine and Pharmacy (MOE), School of Pharmacy, Naval Medical University (Second Military Medical University), Shanghai 200433, China
[3] General Hospital of Central Theater Commond, Wuhan 430070, China
* Correspondence: yuscc1008@163.com (S.Y.); chenxin_0001@126.com (X.C.); canhuizheng@smmu.edu.cn (C.Z.)

Figure S1. NMR spectra of compound D14

^{1}HNMR

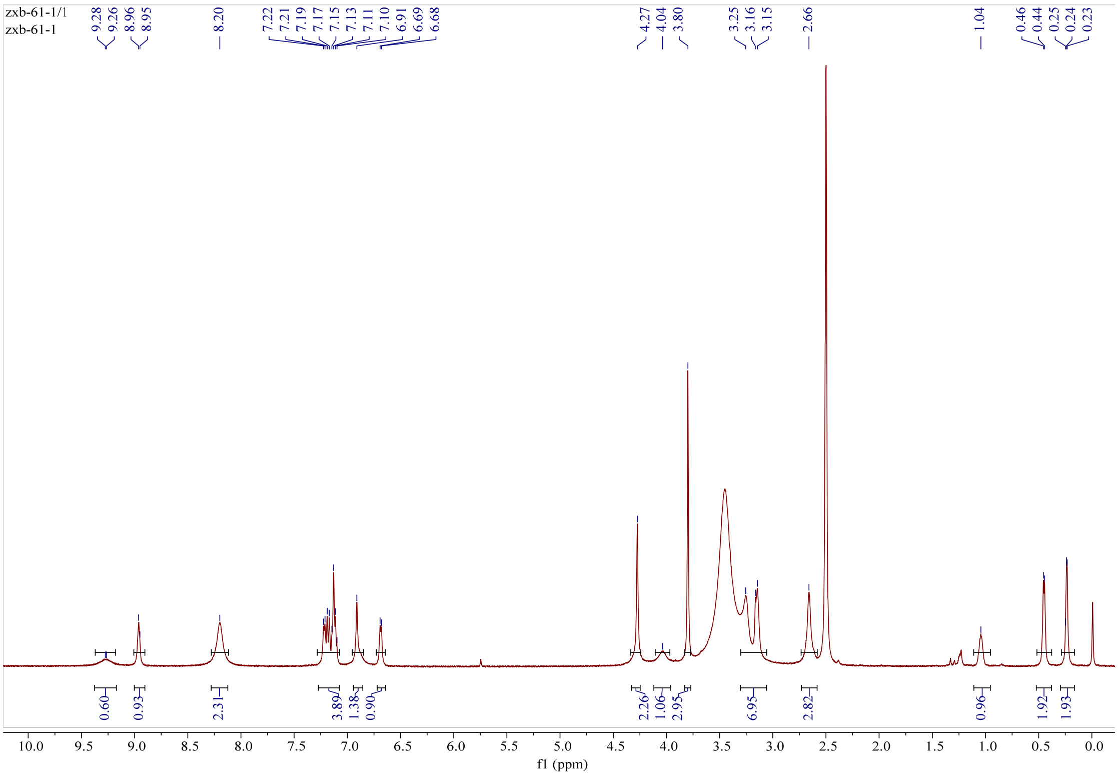

Figure S2. NMR spectra of compound D17

^{1}HNMR

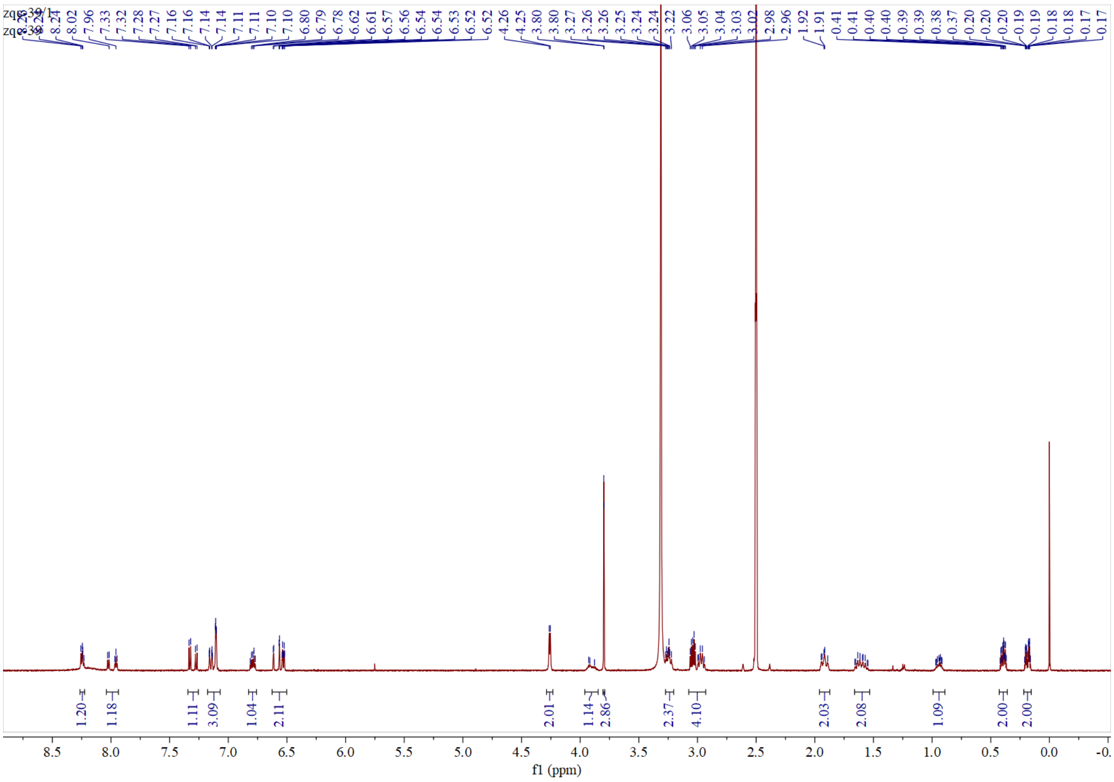

Figure S3. NMR spectra of compound D15

^{1}HNMR

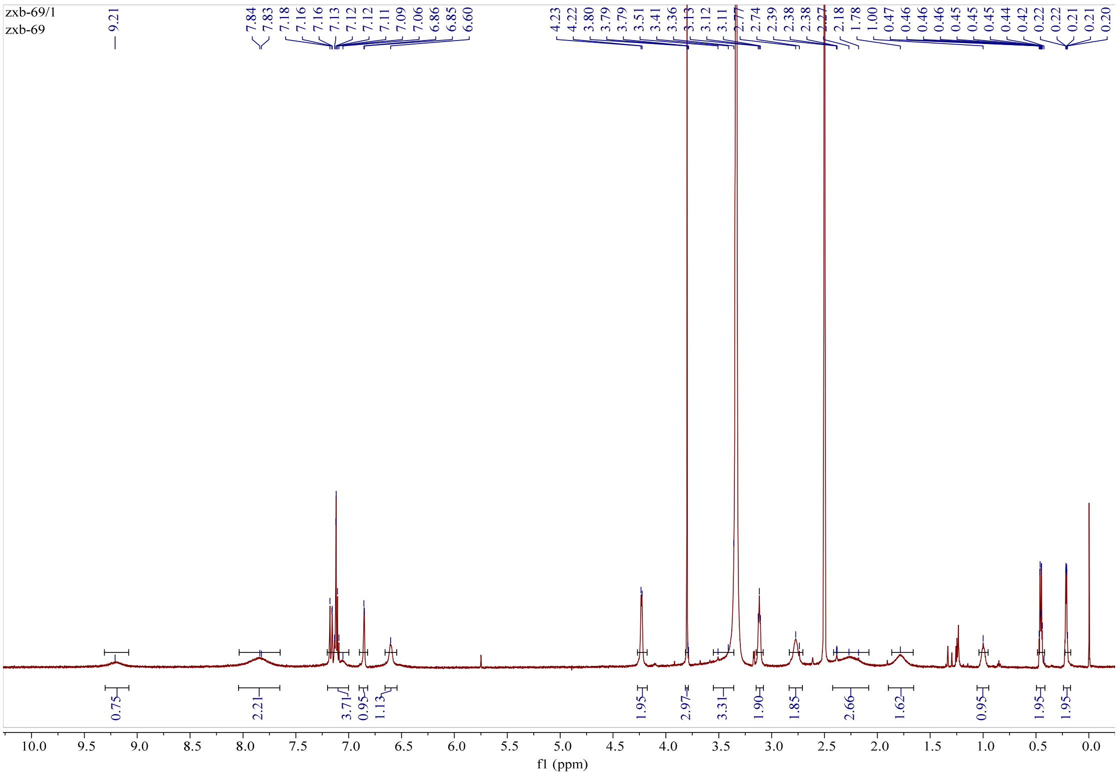

Figure S4. NMR spectra of compound D11

^{1}HNMR

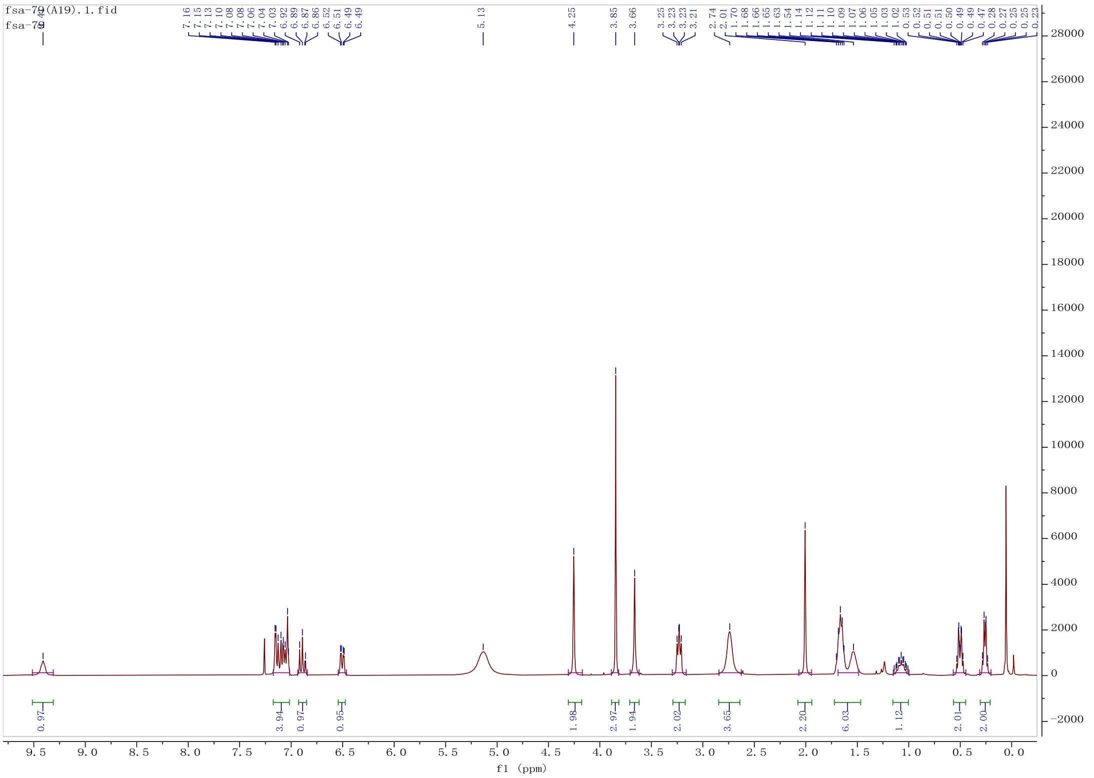

Figure S5. NMR spectra of compound D12

^{1}HNMR

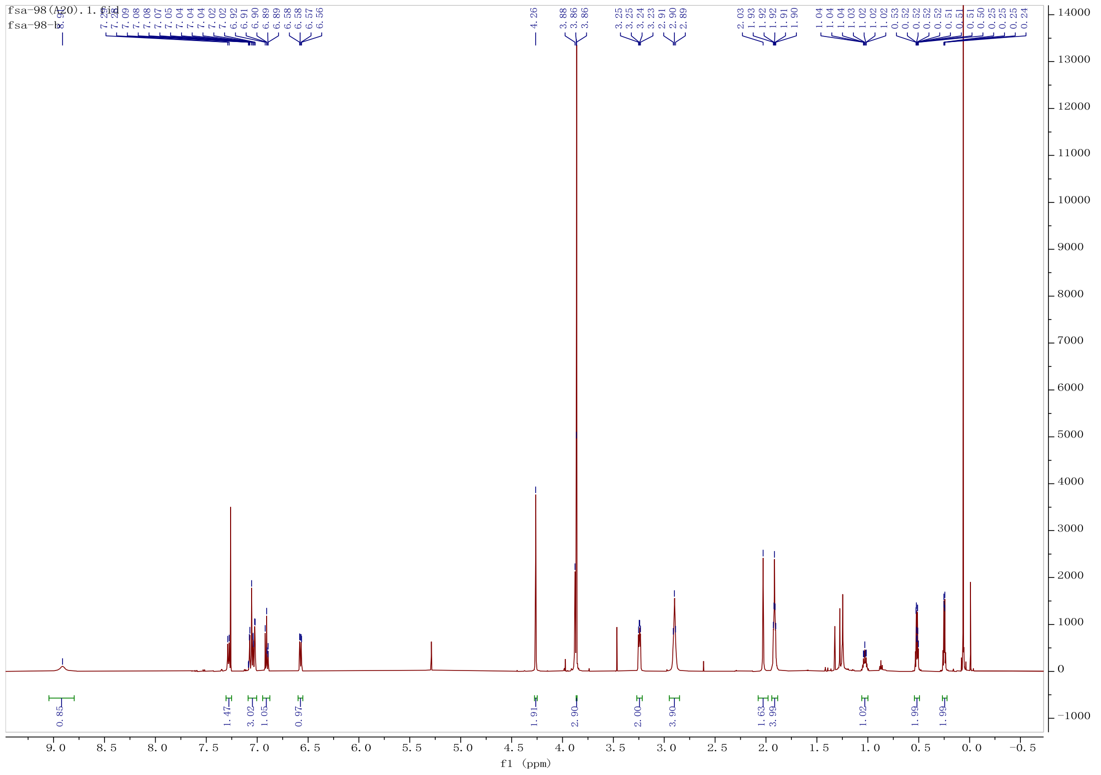

Figure S6. NMR spectra of compound D10

^{1}HNMR

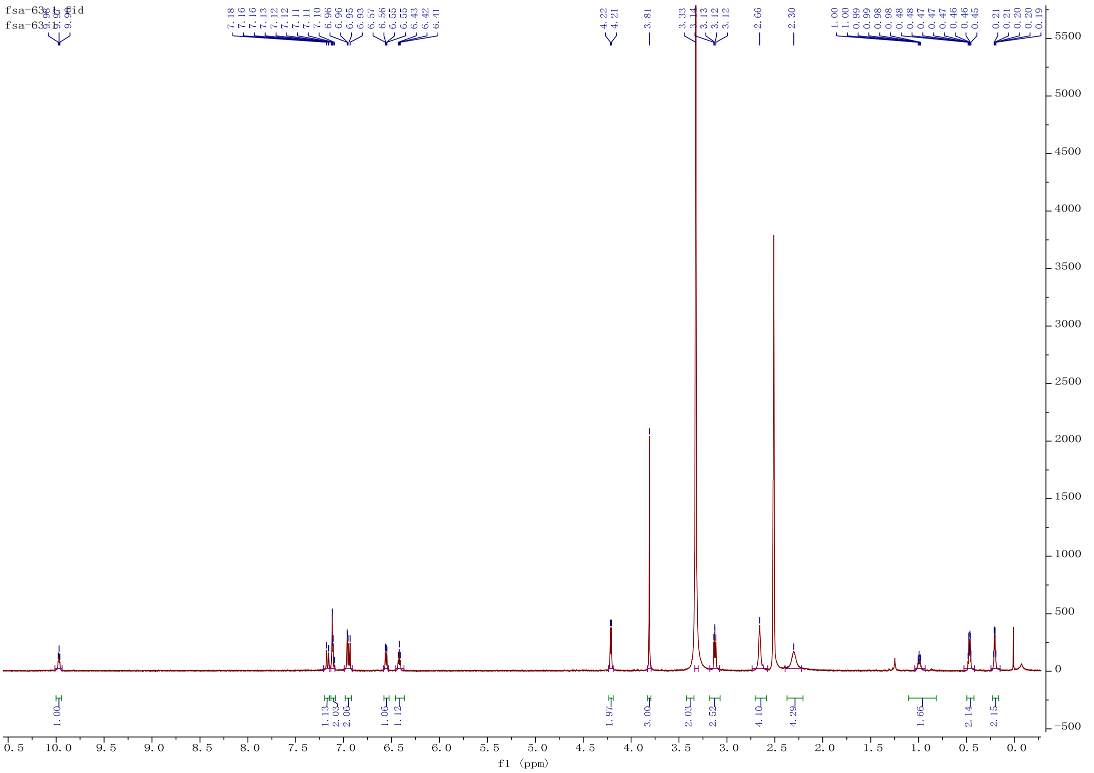

^{13}CNMR

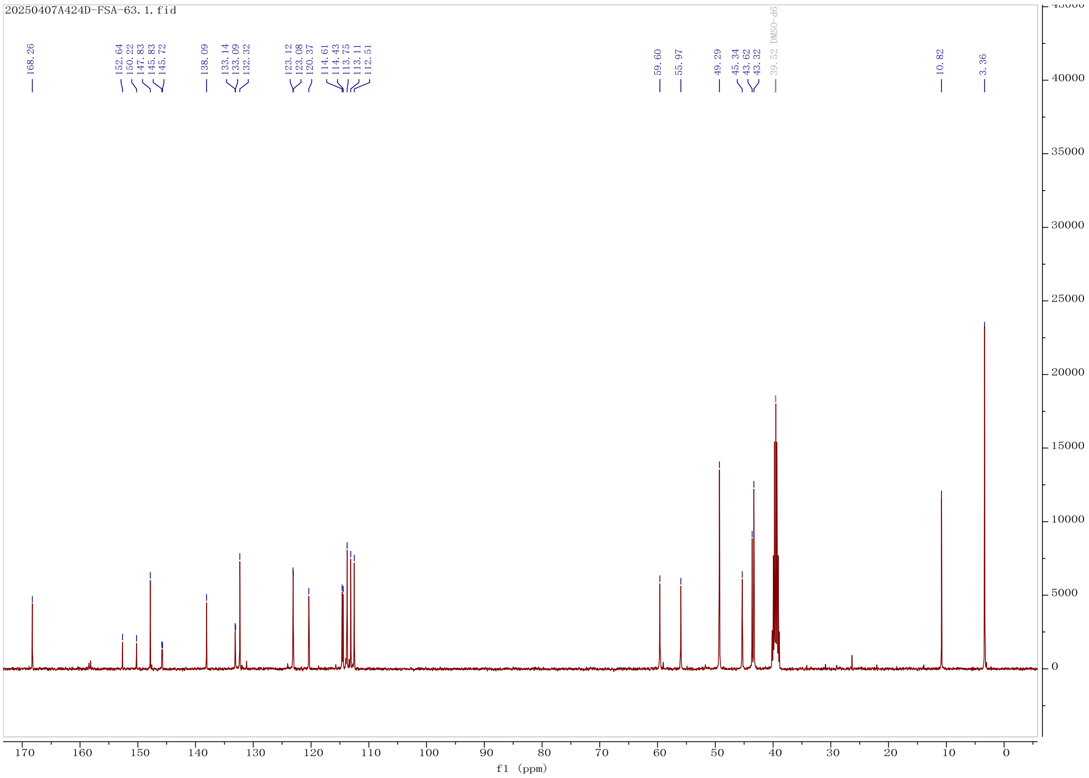
20250407A424D-FSA-63.1.fid
168.26
152.64
150.22
147.83
145.83
145.72
138.09
133.14
133.09
132.32
123.12
123.08
120.37
114.61
114.43
113.75
113.11
112.51
59.60
55.97
49.29
45.34
43.62
43.32
39.52 DMSO-d6
10.82
3.36
f1 (ppm)

Figure S7. NMR spectra of compound D16

¹HNMR

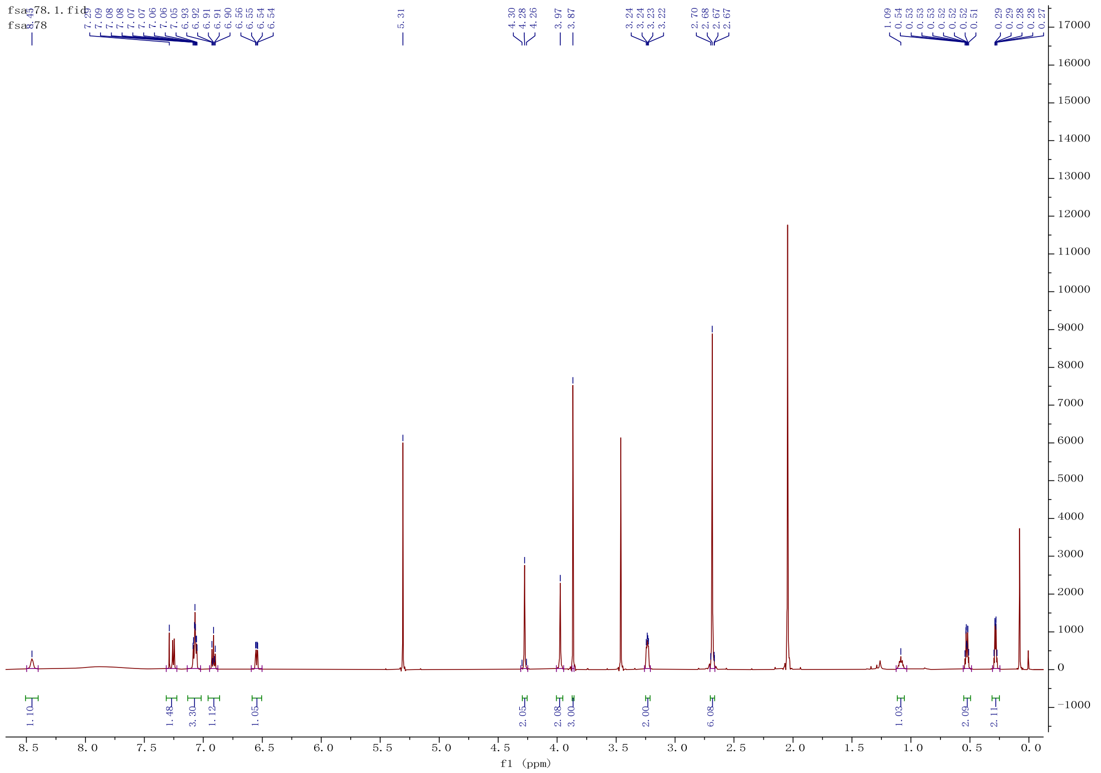

Figure S8. NMR spectra of compound D23

^{1}HNMR

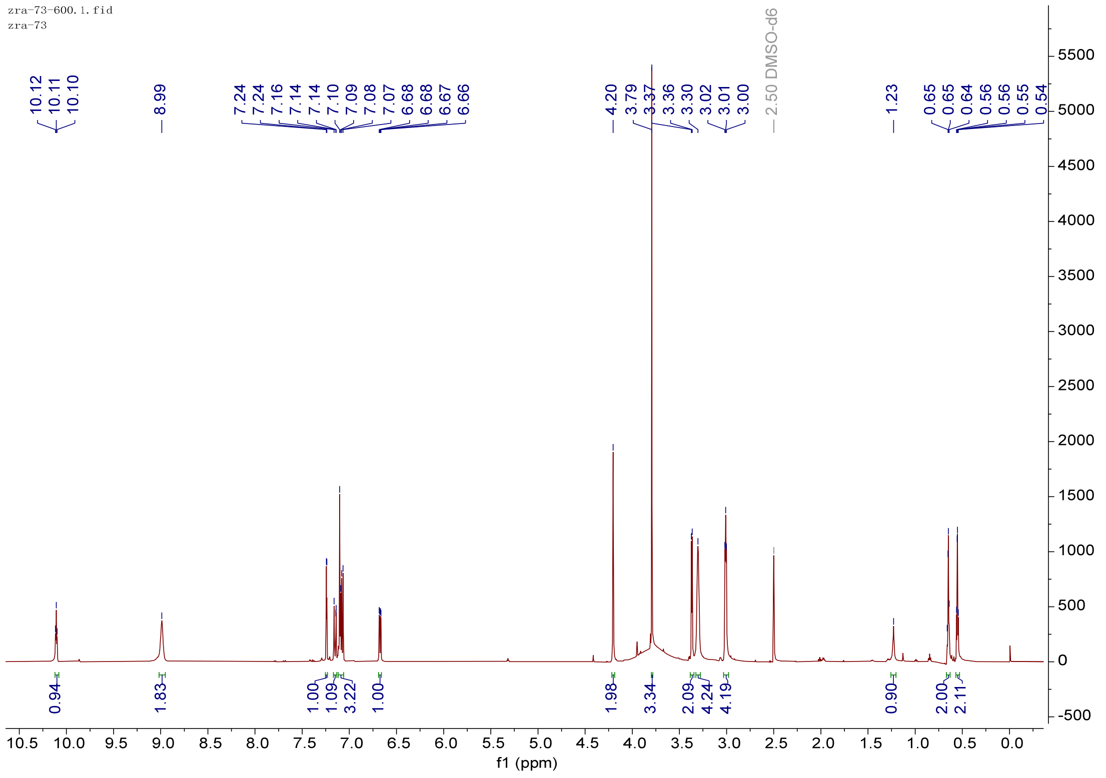

Figure S9. NMR spectra of compound D24

^{1}HNMR

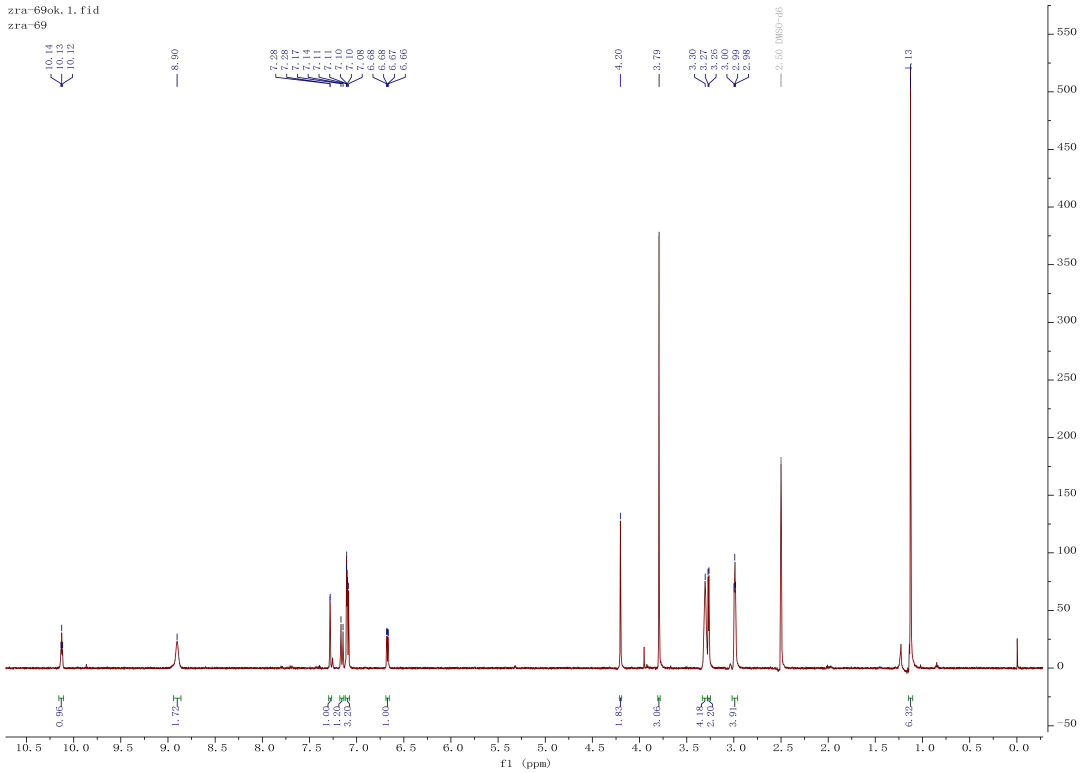

Figure S10. NMR spectra of compound D25

^{1}HNMR

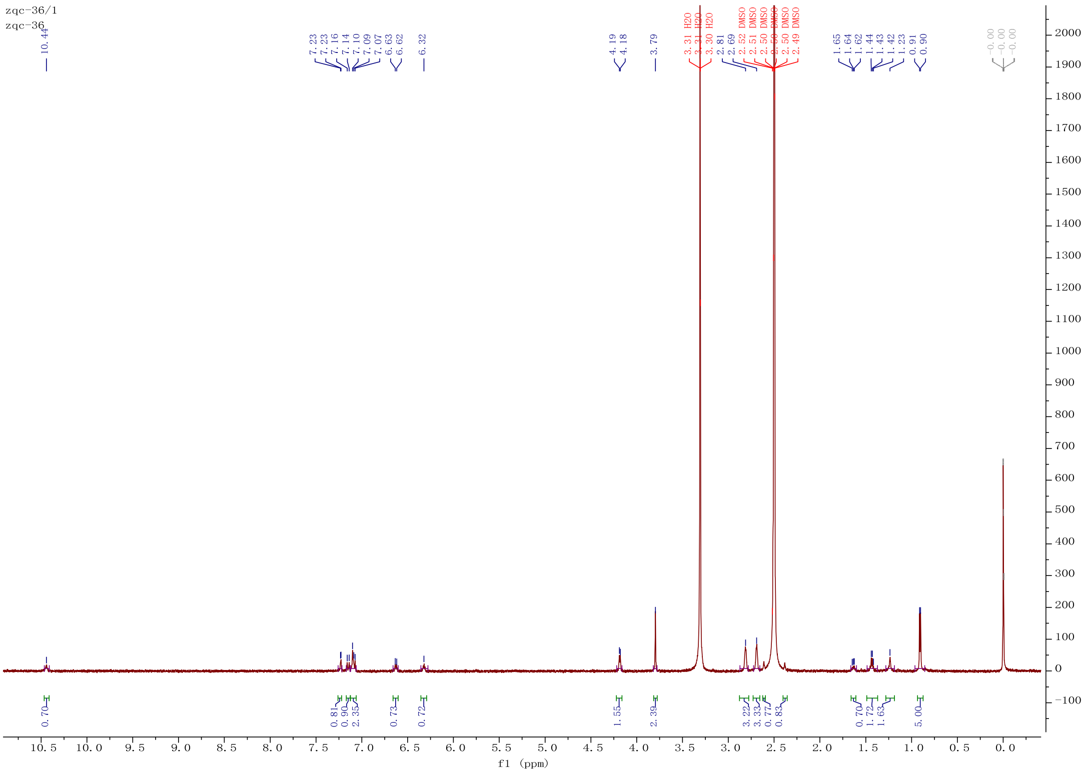

Figure S11. NMR spectra of compound D9

^{1}HNMR

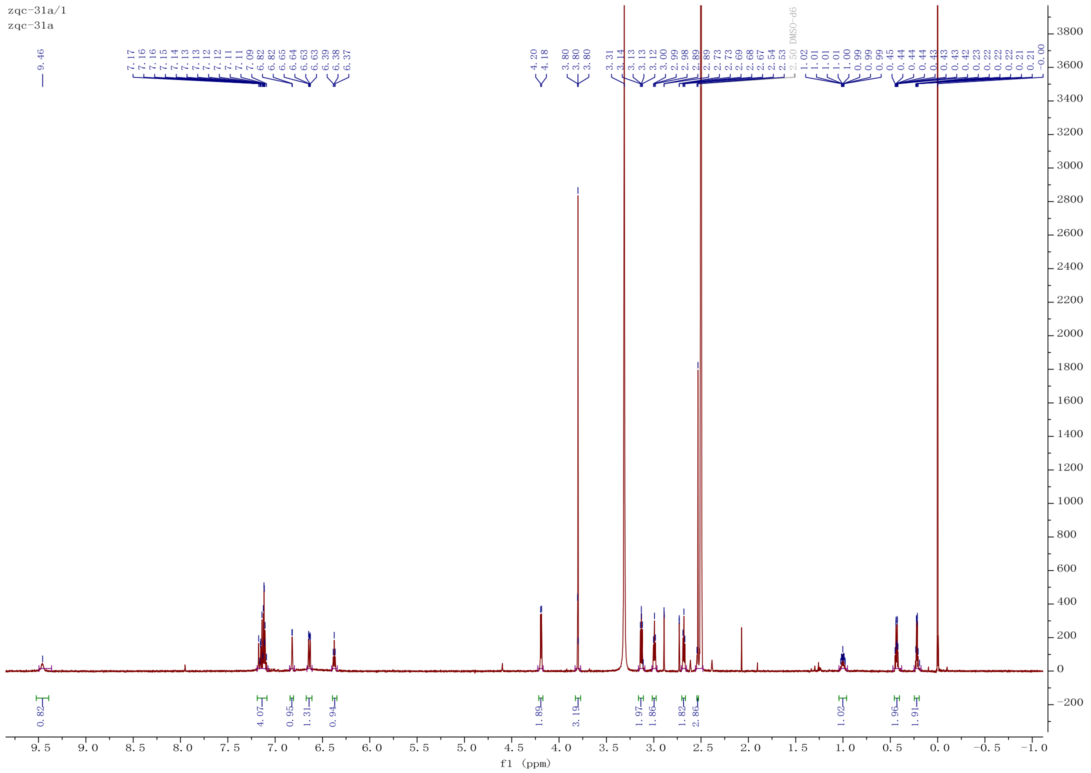

Figure S12. NMR spectra of compound D5

1HNMR

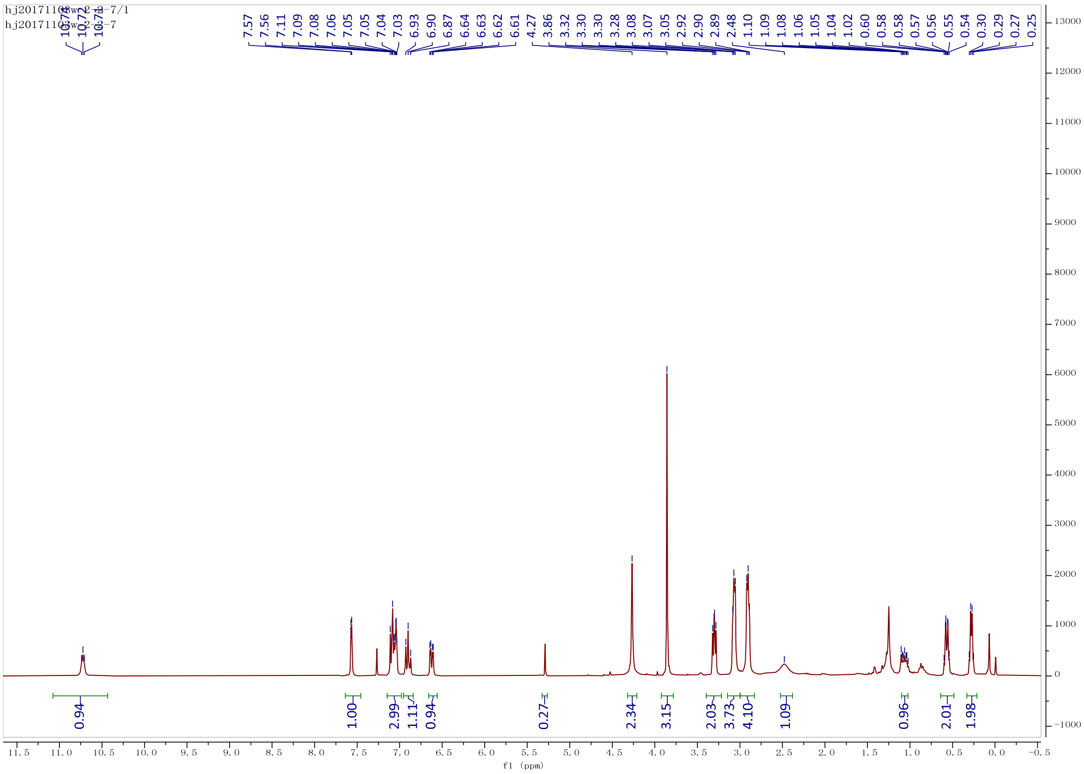

^{13}CNMR

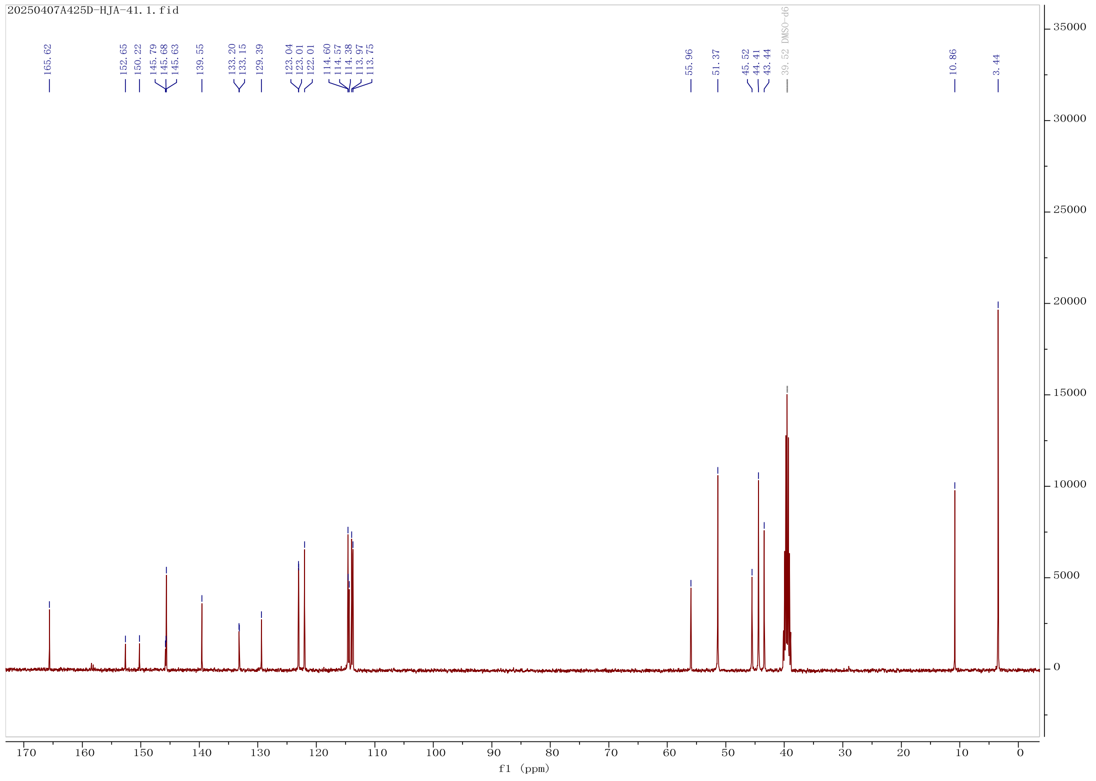
20250407A425D-HJA-41.1.fid
165.62
152.65
150.22
145.79
145.68
145.63
139.55
133.20
133.15
129.39
123.04
123.01
122.01
114.60
114.57
114.38
113.97
113.75
55.96
51.37
45.52
44.41
43.44
39.52 DMSO-d6
10.86
3.44
f1 (ppm)

Figure S13. NMR spectra of compound D26

^{1}HNMR

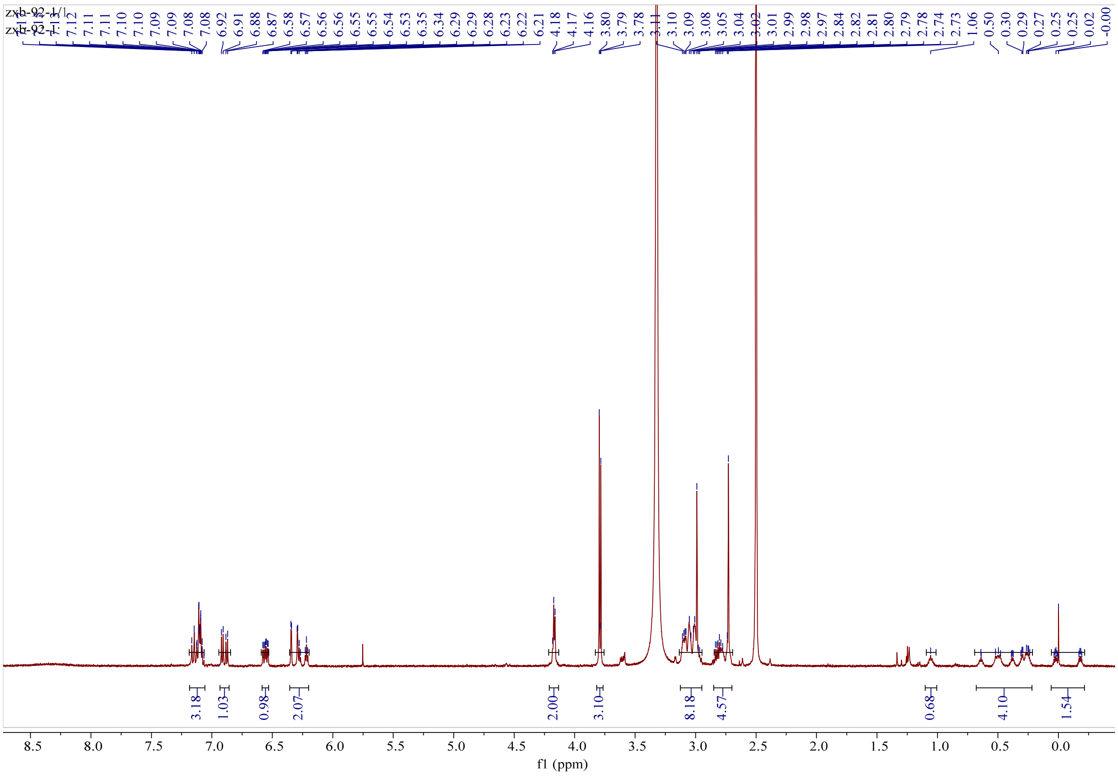

Figure S14. NMR spectra of compound D8

^{1}HNMR

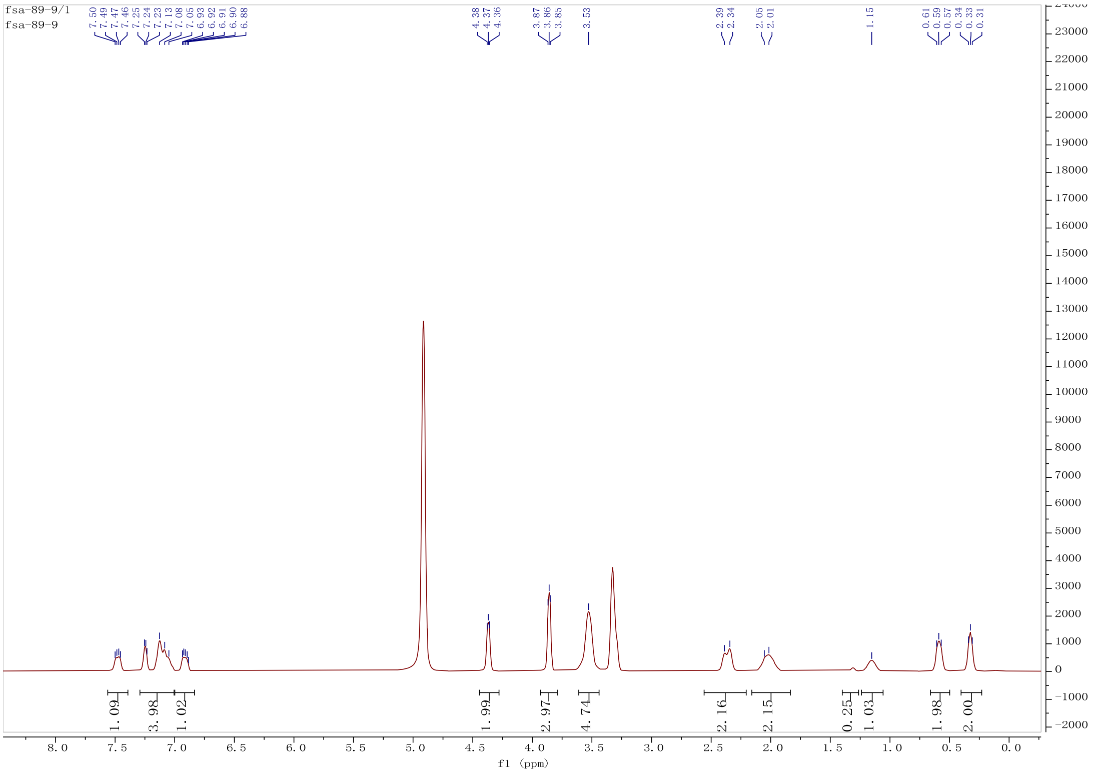

Figure S15. NMR spectra of compound D4

¹HNMR

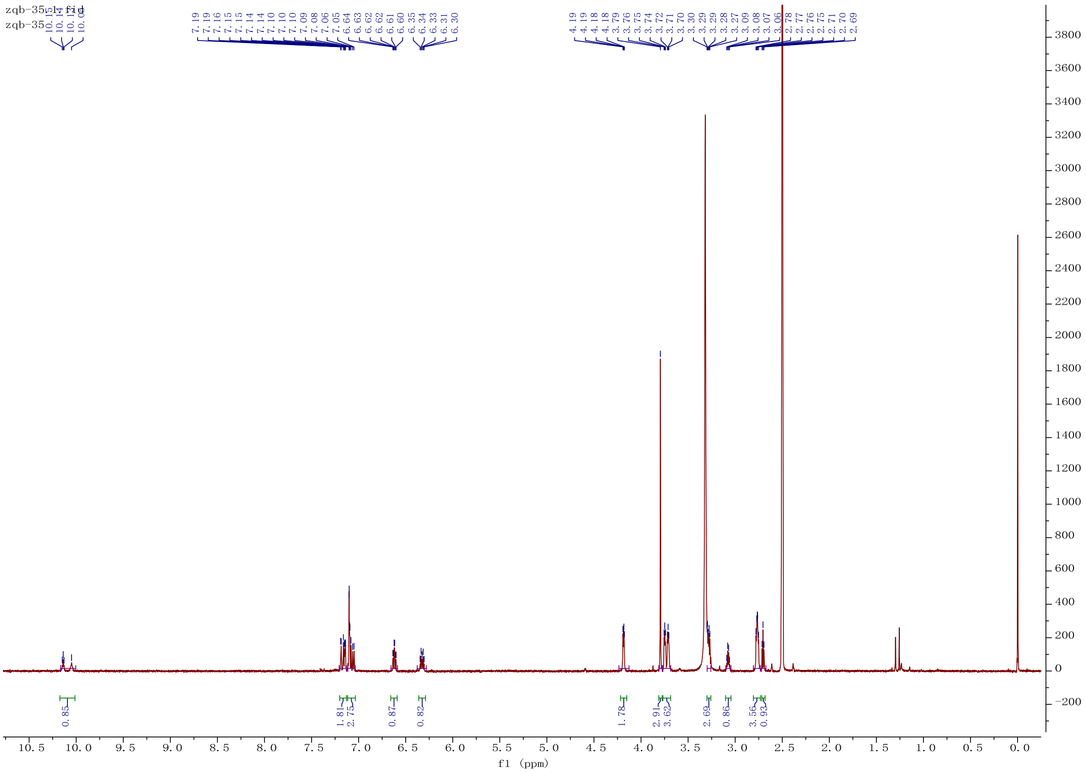

Figure S16. NMR spectra of compound D1

^{1}HNMR

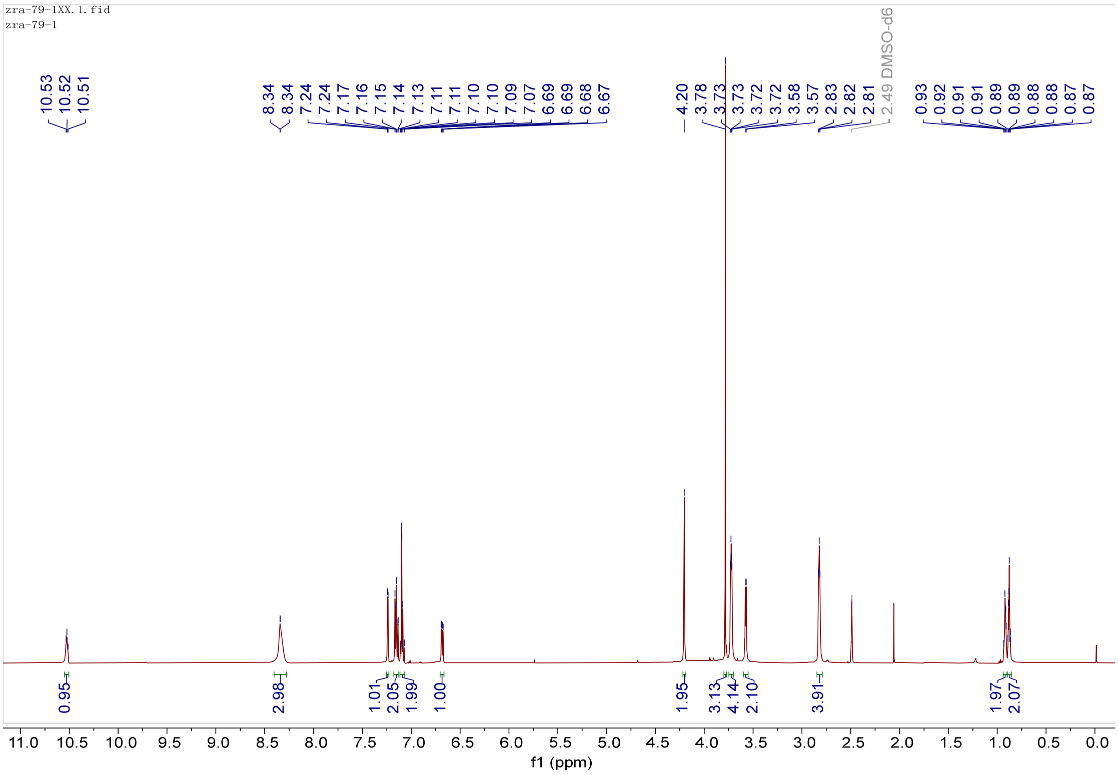

^{13}CNMR

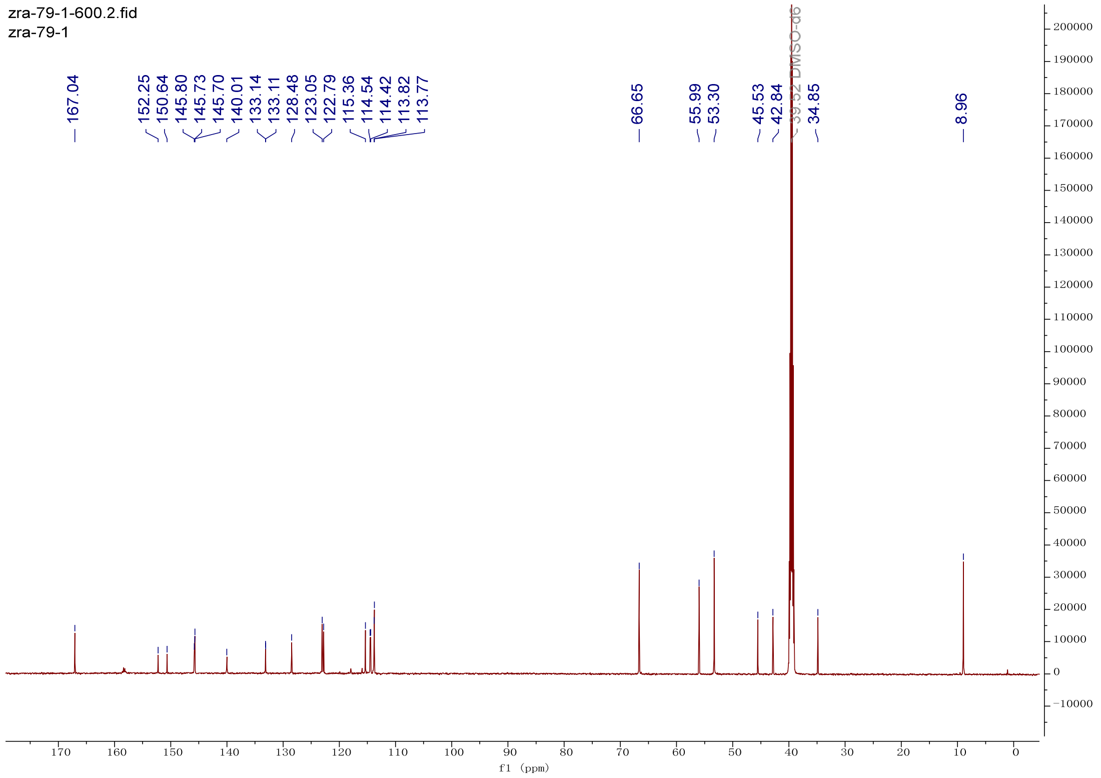

zra-79-1-600.2.fid
zra-79-1
167.04
152.25
150.64
145.80
145.73
145.70
140.01
133.14
133.11
128.48
123.05
122.79
115.36
114.54
114.42
113.82
113.77
66.65
55.99
53.30
45.53
42.84
39.52 DMSO-d6
34.85
8.96
f1 (ppm)

Figure S17. NMR spectra of compound D2

^{1}HNMR

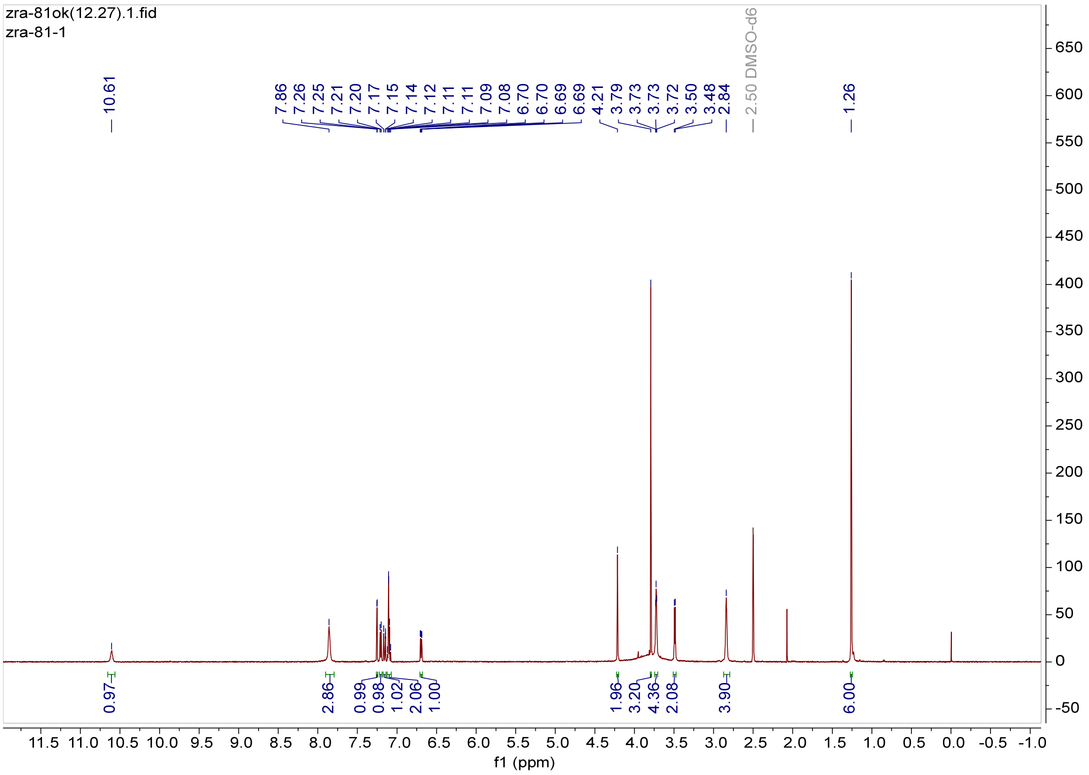

^{13}CNMR

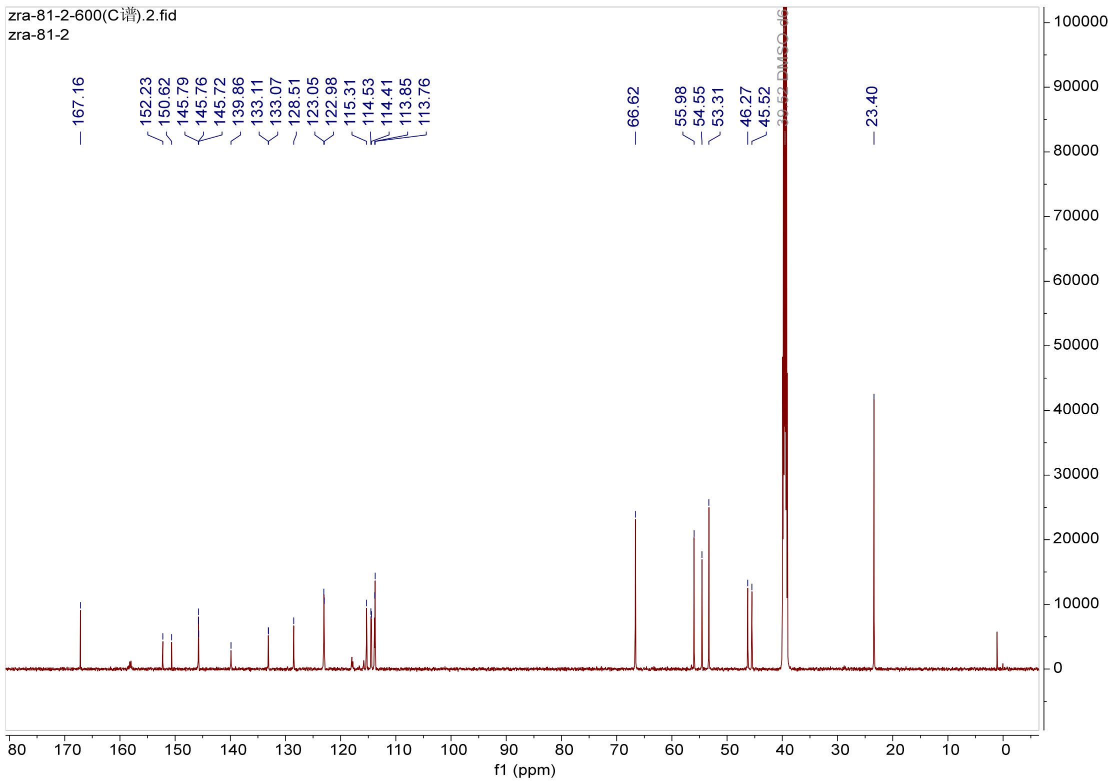

zra-81-2-600(C谱).2.fid
zra-81-2
167.16
152.23
150.62
145.79
145.76
145.72
139.86
133.11
133.07
128.51
123.05
122.98
115.31
114.53
114.41
113.85
113.76
66.62
55.98
54.55
53.31
46.27
45.52
DMSO-d6
23.40
f1 (ppm)

Figure S18. NMR spectra of compound D3

^{1}HNMR

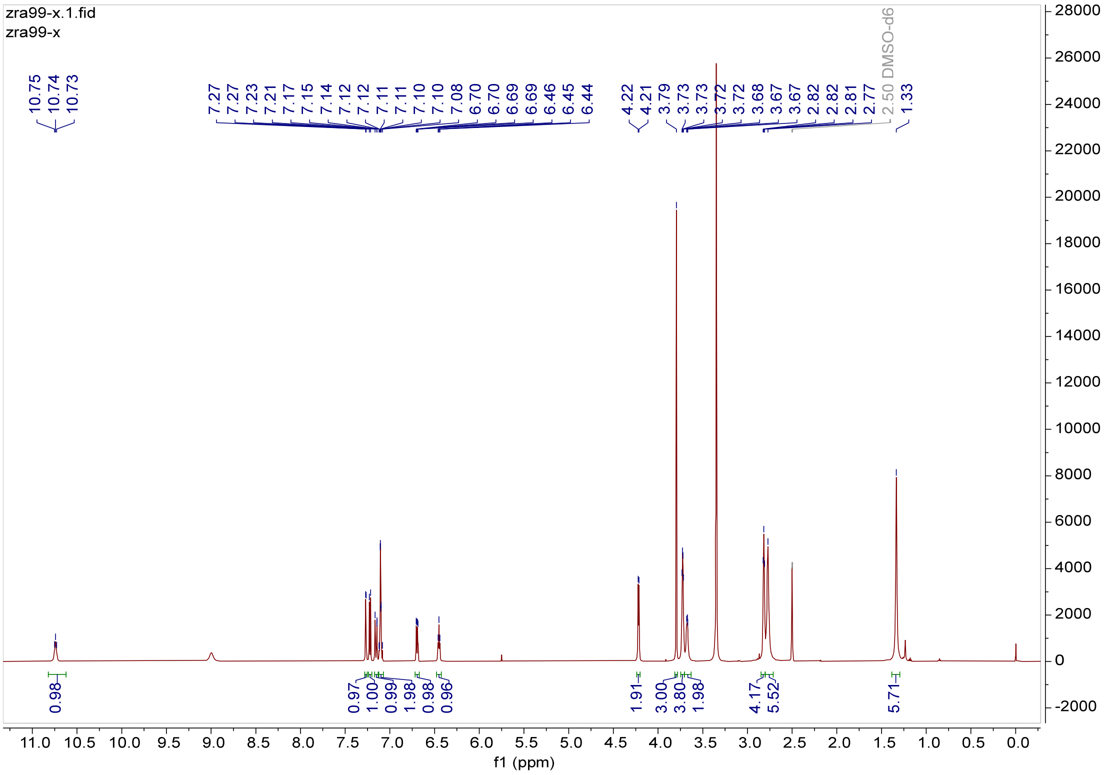

Figure S19. NMR spectra of compound D6

^{1}HNMR

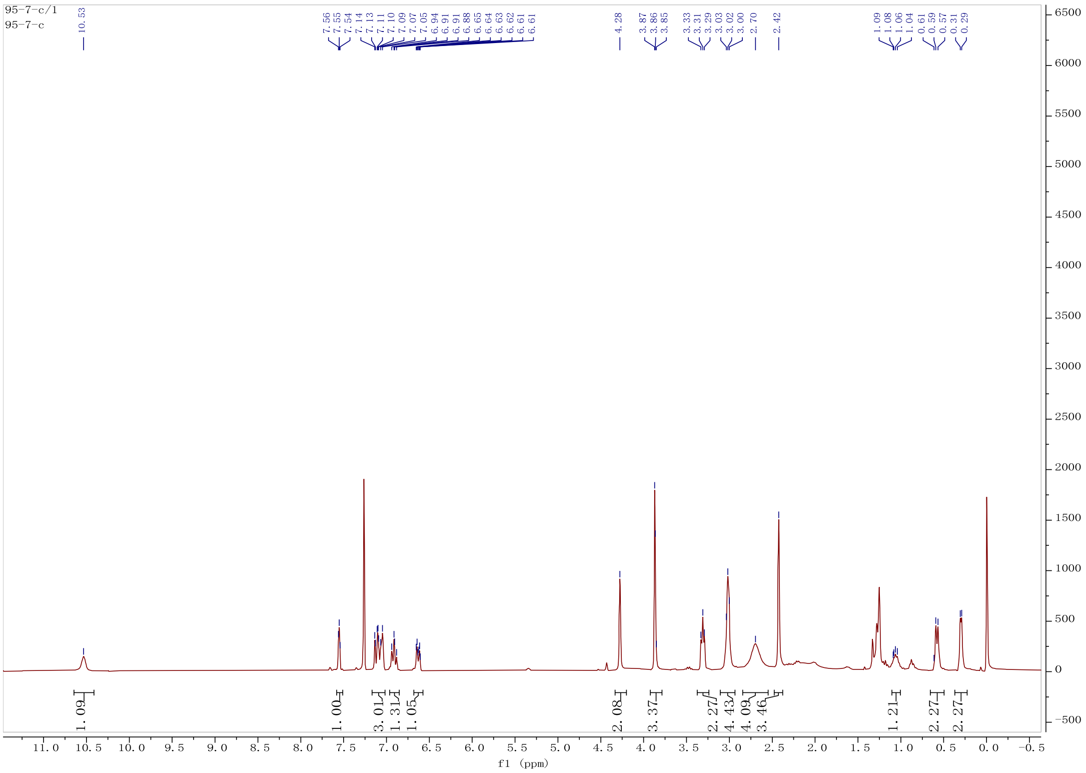

Figure S20. NMR spectra of compound D7

¹HNMR

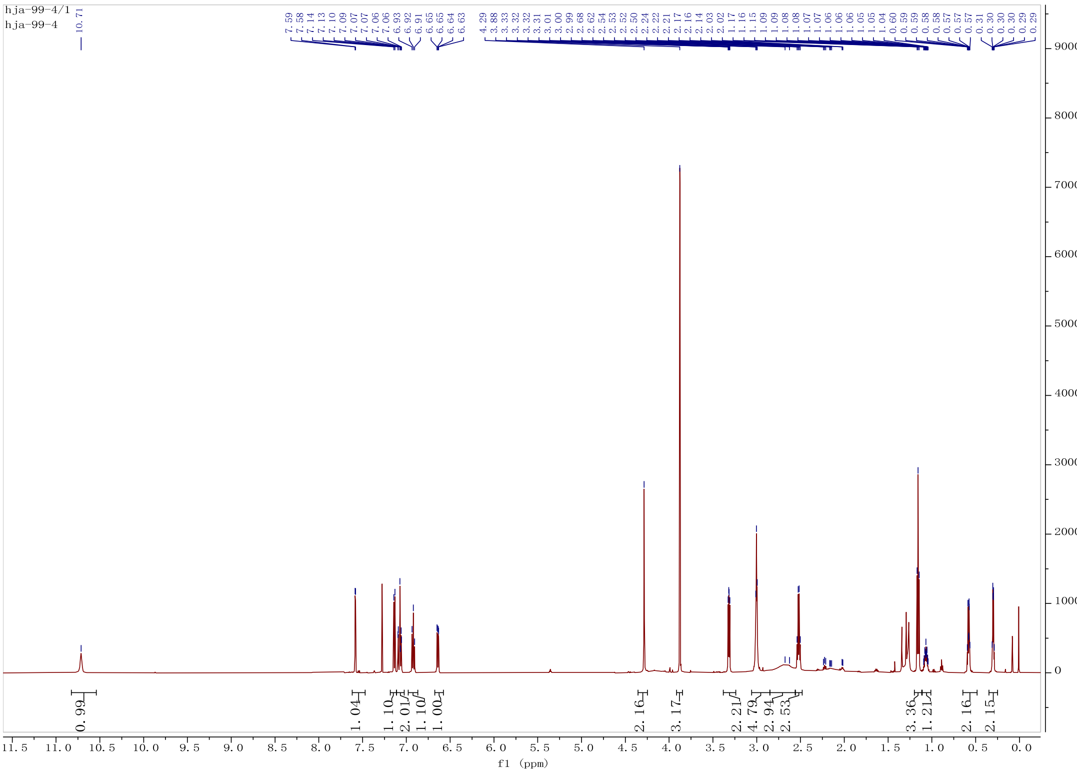

Figure S21. NMR spectra of compound D18

^{1}HNMR

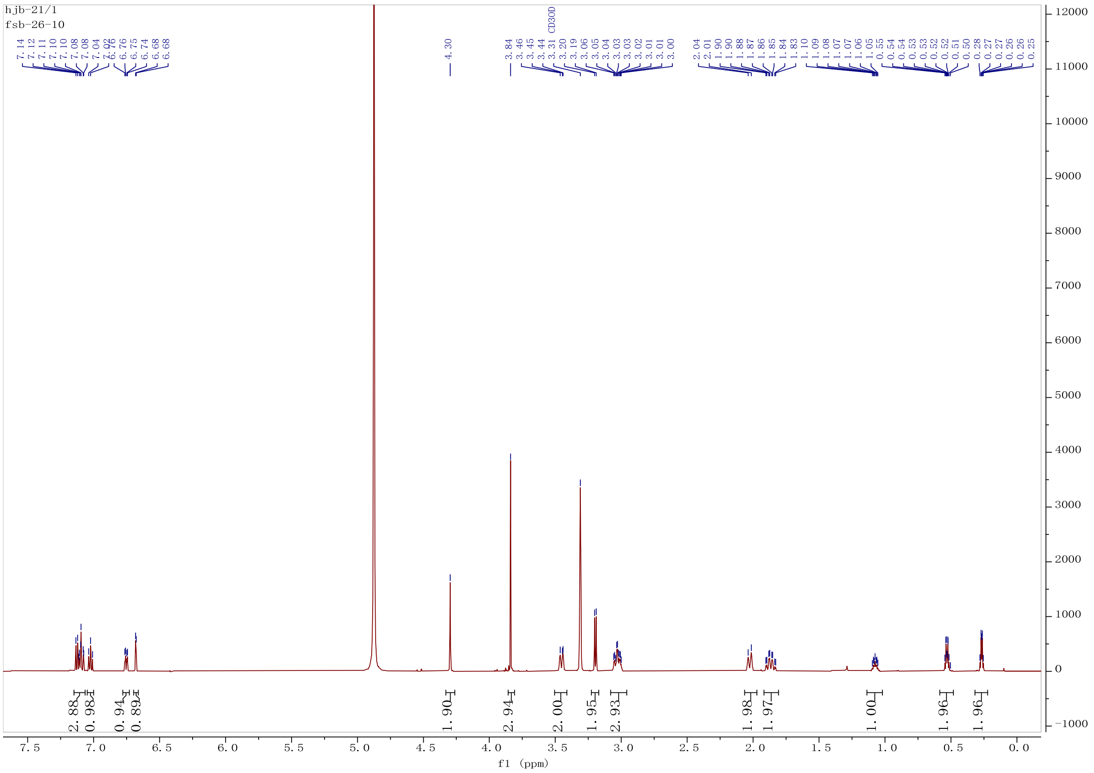

Figure S22. NMR spectra of compound D21

^{1}HNMR

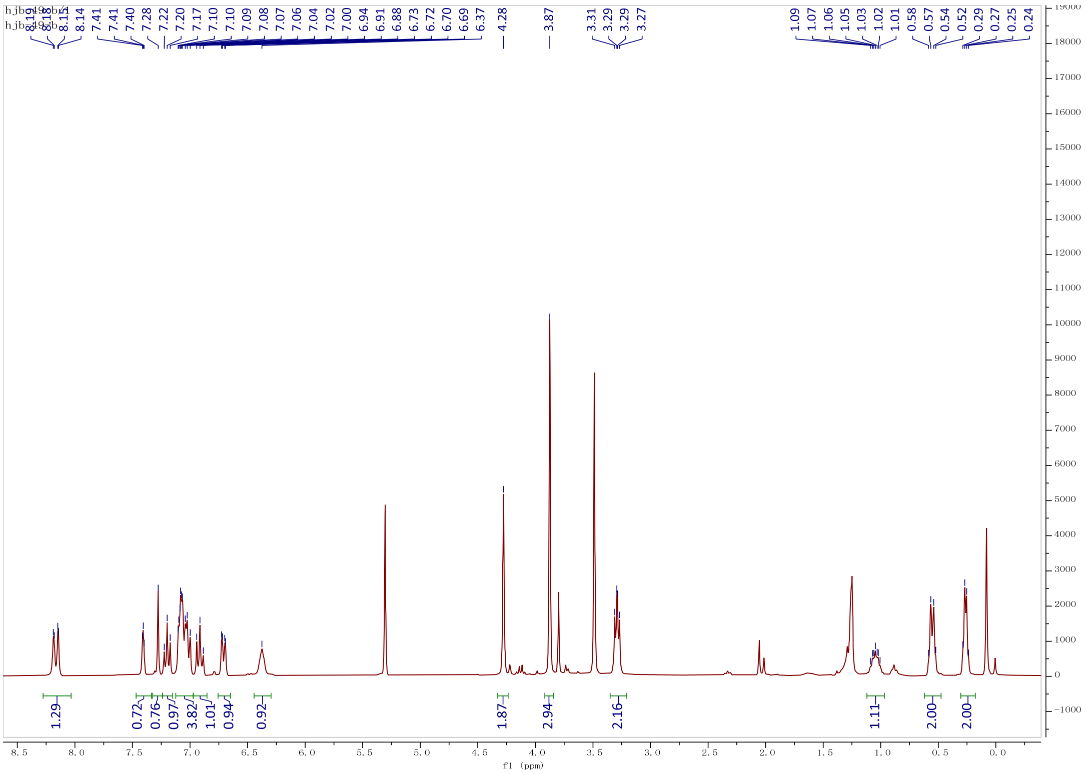

Figure S23. NMR spectra of compound D19

^{1}HNMR

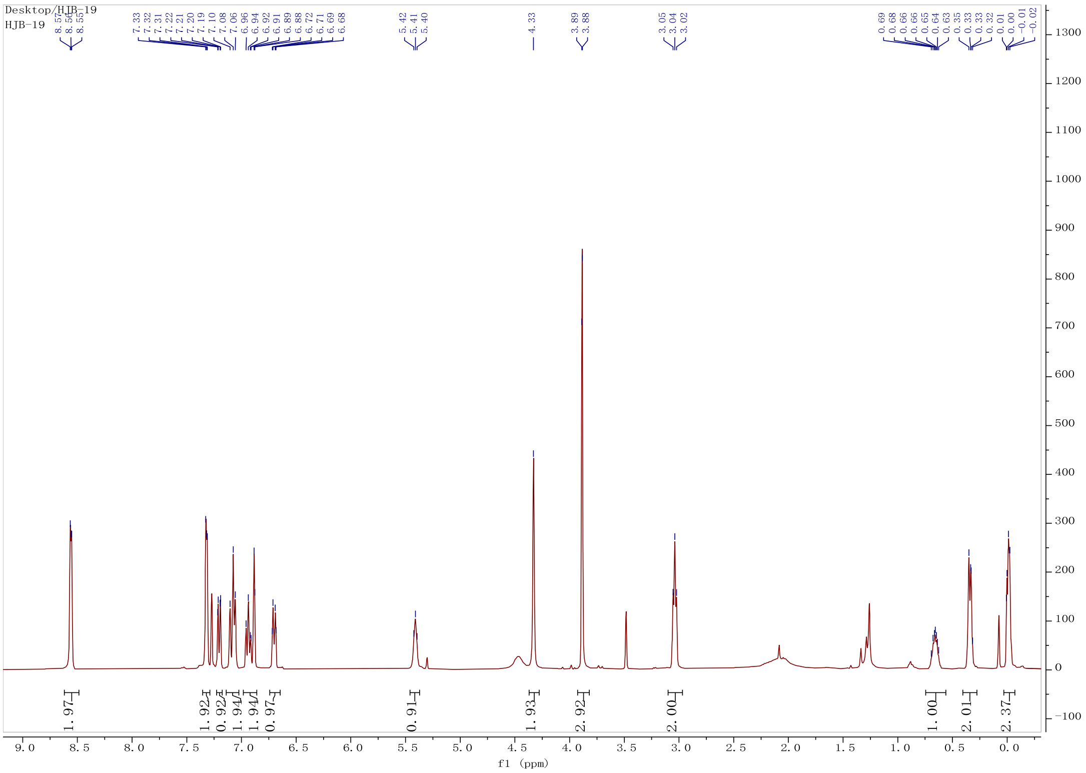

Figure S24. NMR spectra of compound D20

^{1}HNMR

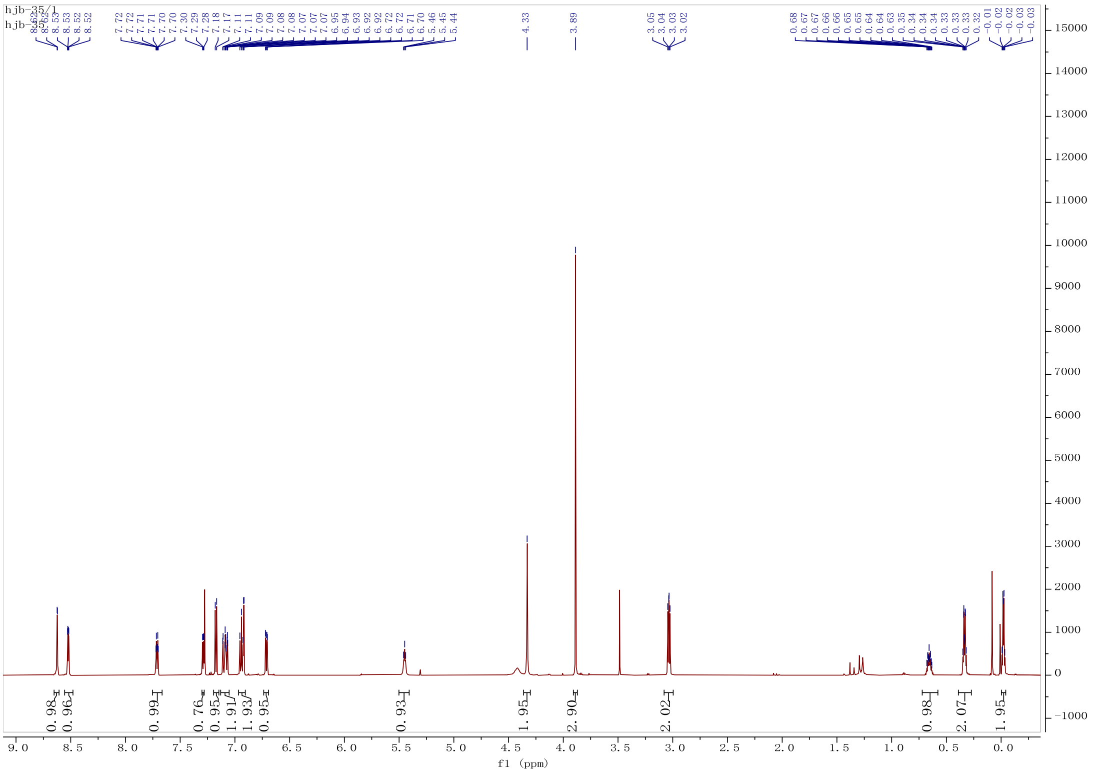

Table S1. Summary of the interactions between C6 and D10 and ALDH2

ALDH2	D10	C6
Hydrogen bond interactions (Asp123)	Piperazine group	/
Hydrogen bond interactions (Asp457)	Amino group of benzylamino moiety	amino group of benzylamino moiety
Hydrogen bond interactions (Phe459)	Carbonyl group	Carbonyl group
Pi-Pi interactions (Phe170, Phe296, Phe459)	Benzene ring of benzylamino moiety	Benzene ring of benzylamino moiety
Hydrophobic interaction (Val120, Cys128, Leu173, Met174, Trp177, Phe292, Phe296, Cys301, Gly460, Ala461, Gln462)	cyclopropylmethyl group	cyclopropylmethyl group
Goldscore	72.92	68.38
